# Computational modeling of ventricular-ventricular interactions suggest a role in clinical conditions involving heart failure

**DOI:** 10.3389/fphys.2023.1231688

**Published:** 2023-09-06

**Authors:** Salla M. Kim, E. Benjamin Randall, Filip Jezek, Daniel A. Beard, Naomi C. Chesler

**Affiliations:** ^1^ Department of Biomedical Engineering, Edwards Lifesciences Foundation Cardiovascular Innovation and Research Center, University of California Irvine, Irvine, CA, United States; ^2^ Department of Molecular and Integrative Physiology, University of Michigan, Ann Arbor, MI, United States; ^3^ Department of Pathological Physiology, First Faculty of Medicine, Charles University, Prague, Czechia

**Keywords:** ventricular interdependence, right ventricular dysfunction, systolic dysfunction, diastolic dysfunction, heart failure with preserved ejection fraction, computational modeling

## Abstract

**Introduction:** The left (LV) and right (RV) ventricles are linked biologically, hemodynamically, and mechanically, a phenomenon known as ventricular interdependence. While LV function has long been known to impact RV function, the reverse is increasingly being realized to have clinical importance. Investigating ventricular interdependence clinically is challenging given the invasive measurements required, including biventricular catheterization, and confounding factors such as comorbidities, volume status, and other aspects of subject variability.

**Methods:** Computational modeling allows investigation of mechanical and hemodynamic interactions in the absence of these confounding factors. Here, we use a threesegment biventricular heart model and simple circulatory system to investigate ventricular interdependence under conditions of systolic and diastolic dysfunction of the LV and RV in the presence of compensatory volume loading. We use the end-diastolic pressure-volume relationship, end-systolic pressure-volume relationship, Frank Starling curves, and cardiac power output as metrics.

**Results:** The results demonstrate that LV systolic and diastolic dysfunction lead to RV compensation as indicated by increases in RV power. Additionally, RV systolic and diastolic dysfunction lead to impaired LV filling, interpretable as LV stiffening especially with volume loading to maintain systemic pressure.

**Discussion:** These results suggest that a subset of patients with intact LV systolic function and diagnosed to have impaired LV diastolic function, categorized as heart failure with preserved ejection fraction (HFpEF), may in fact have primary RV failure. Application of this computational approach to clinical data sets, especially for HFpEF, may lead to improved diagnosis and treatment strategies and consequently improved outcomes.

## 1 Introduction

While the left (LV) and right (RV) ventricles are distinct embryologically, structurally, and functionally (Golob, Moss, and Chesler, 2014), they are linked by a pericardium, shared myofibers, an interventricular septum with common conduction pathway, and a closed loop hemodynamic circuit. The shared myofibers transverse both ventricles, mechanically linking contractile function, termed systolic interdependence (Damiano et al., 1991; Schwarz et al., 2013; Naeije and Badagliacca, 2017; Berglund, Piña, and Herrera, 2020). Experimental studies demonstrate the ability of LV systolic dysfunction (SD) to cause RV SD (Santamore et al., 1976; Damiano et al., 1991). Diastolic interdependence becomes evident when volume overload of one ventricle alters septal dynamics and impedes filling of the other ventricle (Ventetuolo and Klinger, 2014; Naeije and Badagliacca, 2017; Berglund, Piña, and Herrera, 2020). The resultant upward and leftward shift in the end-diastolic pressure-volume relationship (EDPVR) can be misinterpreted as chamber or free wall stiffening. While systolic and diastolic interdependence have been investigated for over a century (Henderson and Prince, 1914), the individual contributions of the pericardium, shared myofibers, septum, and hemodynamic circuit to these phenomena, particular during heart failure (HF), remain incompletely understood.

Heart failure with reduced ejection fraction (HFrEF; LV ejection fraction <50%) represents nearly 50% of heart failure cases worldwide and is characterized by LV SD (Murphy, Ibrahim, and Januzzi, 2020). The impact of this LV SD on systemic hemodynamics is often so severe that any change in RV function is ignored clinically. However, secondary RV dysfunction is common, and metrics of RV function, such as cardiac power output (CPO) (Yildiz and Yenigun, 2021), can predict outcomes in HFrEF (Bosch et al., 2017). In heart failure with preserved ejection fraction (HFpEF), which represents the other nearly 50% of HF cases worldwide (Pfeffer, Shah, and Borlaug 2019) and is highly heterogenous (Gorter, Rienstra, and van Veldhuisen, 2017; Jones et al., 2021), there is debate regarding whether LV diastolic dysfunction (DD) (Lekavich et al., 2015), RV SD or DD (Berglund, Piña, and Herrera, 2020; Kadry et al., 2020), or biventricular dysfunction (Rommel et al., 2018) is the major contributor. In both HFrEF and HFpEF, RV function is important to exercise capacity (Sharma and Kass, 2014; Vonk et al., 2019; Santens et al., 2020; Comunale et al., 2021). Thus, interventricular interactions are critical to both quality of life and outcomes in LV failure. Similarly, in primary RV failure, due to, e.g., pulmonary arterial hypertension, LV mechanics are affected (Stojnic et al., 1992; Gan et al., 2006; Puwanant et al., 2010; Hardegree et al., 2013). Thus, knowledge gaps exist for ventricular-ventricular interactions (VVI) in all types of HF, including LV SD, LV DD, RV SD, and RV DD.

Preclinical studies have addressed some of these knowledge gaps. Early isolated heart studies in large animals showed that due to VVI, LV contraction contributes to 60%–70% of RV systolic function (Santamore et al., 1976; Damiano et al., 1991). Ischemic damage to the RV free wall has been shown to impair LV filling in mice (Sicard et al., 2019) and LV ejection in swine (Brookes et al., 1999). In the former rodent studies, LV DD was caused by septal hypertrophy and flattening; in the latter swine studies, LV SD appeared secondary to impaired filling due to pericardial constraints. In a high-fat diet rodent model of HFpEF, significant biventricular dysfunction was found, where RV dysfunction (mildly reduced ejection fraction) was attributed to RV hypertrophy and changes in myofilament sensitivity (Hegemann et al., 2021).

Computational modeling is aptly positioned for investigating VVI because the influence of specific contributors to these interactions can be interrogated individually. For example, the impact of the pericardium, shared myofibers, interventricular septum, and LV and RV preload and afterload as determined by the characteristics of the cardiovascular system (CVS) can be varied independently. In addition, the absence of subject variability and potential species differences simplifies interpretation. Numerous CVS models have been used to explore ventricular dysfunction; however, few account for VVI without computationally and time intensive finite element modeling. Additionally, volume loading as a surrogate for HF compensation is computationally expensive with high order models. Thus, we employ the Lumens et al. three-segment (TriSeg) biventricular model of the heart (2009) because it accounts for differences in RV and LV geometry and captures VVI through shared myofibers and the septum while remaining computationally simple. Previously this model has been used to investigate biventricular mechanics including septal dynamics in primary RV dysfunction due to pulmonary hypertension and pulmonary arterial hypertension (Lumens et al., 2009; Palau-Caballero et al., 2017).

To develop a predictive mechanistic understanding of interventricular interactions under conditions of HFrEF, HFpEF, and heart failure with moderately reduced ejection fraction (HFmrEF) with compensatory volume loading, we combine the TriSeg model with a closed-loop lumped-parameter circulation model. Our simulations reveal that: 1) RV SD and DD induce an apparent LV stiffening that is due to VVI and not to intrinsic changes in the properties of the LV or pericardial constraints; 2) LV dysfunction causes a compensatory increase in RV CPO to supply sufficient LV preload when volume loading is sufficient to maintain normal systemic pressures; and 3) RV dysfunction contributes to LV dysfunction via impaired Frank-Starling mechanism in the LV. The model codes, which are parameterized to simplify the use of potentially subject-specific data, are freely distributed to facilitate further independent exploration of the mechanisms investigated here.

## 2 Methods

### 2.1 Normative hemodynamic data

We establish a hypothetical ideal subject that represents a healthy adult (weight −70 kg). Normative values for systemic, LV, and RV chamber pressures and volumes as well as total blood volume for this hypothetical subject were based on literature values (Table 1). End-systolic pressure (ESP) for the LV was assumed to be 5% greater than the mean arterial systolic blood pressure ( $\bar{SBP}$ ) since the circulation model does not account for pulse-wave propagation. End-diastolic volume (EDV) (assumed to be the same for the RV and LV) is set arbitrarily within a normal range; end-systolic volumes (ESV) are calculated based on a stroke volume (SV) of 75 mL.

**TABLE 1:** Normative values for a healthy 70 kg adult based on literature values and established relationships.

Symbol	Description	Unit	Value	Source
$\bar{SBP}$	Mean systolic arterial blood pressure	mmHg	120	Vasan et al. (2001)
$\bar{DBP}$	Mean diastolic arterial blood pressure	mmHg	80	Vasan et al. (2001)
${ESP}_{LV}$	LV end-systolic pressure	mmHg	$1.05\times \bar{SBP}$	Lampert (2018)
${EDP}_{LV}$	LV end-diastolic pressure	mmHg	5	BOUCHARD et al. (1971), Landsberg (2018)
${ESV}_{LV}$	LV end-systolic volume	mL	50	Padsalgikar (2017)
${EDV}_{LV}$	LV end-diastolic volume	mL	125	Clay et al. (2006), Padsalgikar (2017)
${ESP}_{RV}$	RV end-systolic pressure	mmHg	25	Lampert (2018)
${EDP}_{RV}$	RV end-diastolic pressure	mmHg	${EDP}_{LV}$ /4	Chemla et al. (2002), Lampert (2018)
${ESV}_{RV}$	RV end-systolic volume	mL	${ESV}_{LV}$	
${EDV}_{RV}$	RV end-diastolic volume	mL	125	
TBV	Total blood volume	mL	5,000	

LV, left ventricle; RV, right ventricle.

### 2.2 Cardiac model parameterization

The TriSeg model is adapted from Lumens et al. (2009) in which the ventricles are constructed from three thick-walled spherical, segments corresponding to the ventricular left wall (LW) and right wall (RW) with a septal wall (SW) in between. To simplify parameterization of the cardiac model for individual subjects, we developed a generalized method in which ESVs and EDVs are used to compute LW, RW, and SW thicknesses. We also used a sinusoidal driving function (Marquis et al., 2018) to modulate length-dependent myocyte contraction instead of the Lumens et al. heuristic force-velocity approach (Lumens et al., 2009). We also simplified the passive stiffness model and included a pericardial constraint as in Jezek et al. (2022). The novel aspects of our approach are detailed below, and a complete description is available in the Supplementary Appendix.

#### 2.2.1 Parameterized reference geometry

In the TriSeg model, the midwall is defined as a theoretical surface radially half-way between the inner and outer surfaces of the ventricular wall (Lumens et al., 2009) (the normal ventricular geometry is illustrated on Figure 4A, where the midwall is the dashed line creating intersection points where the tension is balanced). The midwall reference surface area for the LW, RW, or SW segments, $A_{m,ref}$ , and midwall volume, $V_{m},$ (the volume of the wall from the outer surface to the midwall) are highly influential shape parameters that we parameterize based on input ${EDV}_{LV}$ and ${EDV}_{RV}$ values that could be collected from a subject via magnetic resonance imaging. We begin with a LV that is made up of the LW and SW and assume that the LW and SW have the same wall thickness ( h , such that $h_{LW}=h_{SW}=h_{LV}$ ). With the TriSeg assumption of spherical ventricles, the inner chamber radii $r_{i}$ , are

$$r_{i,LV}={\left(\frac{4}{3\pi }{EDV}_{LV}\right)}^{\frac{1}{3}}\;and\;r_{i,RW}={\left(\frac{4}{3\pi }{EDV}_{RV}\right)}^{\frac{1}{3}},$$ (1)

and given thickness, h , the midwall radii $r_{m}$ , are

$$r_{m,LV}=r_{LV}+\frac{1}{2}h_{LV}\;and\;r_{m,RW}=r_{RW}+\frac{1}{2}h_{RW},$$ (2)

and finally, the outer radii $r_{o}$ , are

$$r_{o,LV}=r_{LV}+h_{LV}\;and\;r_{o,RW}=r_{RW}+h_{RW}.$$ (3)

Midwall surface areas and wall volumes can then be determined from midwall radii as

$$A_{m,ref,LV}=4\pi r_{m,LV}^{2}\;and\;A_{m,ref,RW}=4\pi r_{m,RW}^{2},$$ (4)

and

$$V_{w,LV}=\frac{4}{3}\pi r_{o,LV}^{3}-{EDV}_{LV}\;and\;V_{w,RW}=\frac{4}{3}\pi r_{o,RW}^{3}-{EDV}_{RV}.$$ (5)

We assume the LW is 2/3 and the SW is 1/3 of the entire LW volume, that is,

$$V_{w,LW}=\frac{2}{3}V_{w,LV}\;and\;V_{w,SW}=\frac{1}{3}\;V_{w,LV},$$ (6)

and in terms of EDV,

$$V_{w,LW}=\frac{8\pi }{9}{\left({\left(\frac{4}{3\pi }{EDV}_{LV}\right)}^{\frac{1}{3}}+h_{LV}\right)}^{3}-{\frac{2}{3}EDV}_{LV}\;and\;V_{w,SW}=\frac{4\pi }{9}{\left({\left(\frac{4}{3\pi }{EDV}_{LV}\right)}^{\frac{1}{3}}+h_{LV}\right)}^{3}-{\frac{1}{3}EDV}_{LV}.$$

Similarly, for the midwall reference areas,

$$A_{m,ref,LW}=\frac{2}{3}A_{m,ref,LV}\;and\;A_{m,ref,SW}=\frac{1}{3}\;A_{m,ref,LV}$$ (7)

which are then,

$$A_{m,ref,LW}=\frac{8\pi }{3}{\left(\frac{4}{3\pi }{EDV}_{LV}\right)}^{\frac{1}{3}}+\frac{4\pi }{3}h_{LV}\;and\;A_{m,ref,SW}=\frac{4\pi }{3}{\left(\frac{4}{3\pi }{EDV}_{LV}\right)}^{\frac{1}{3}}+\frac{2\pi }{3}h_{LV}.$$

As indicated, both the SW volume and surface area adopt the TriSeg assumption that the septum shares the same properties as the LW, comprising a third of the LV geometry. With these reference areas and volumes, the process of computing ventricular geometries including curvature, axial position, and radial position follow the method developed by Lumens et al. (2009).

#### 2.2.2 Passive stiffness and contraction

As described by Lumens et al. (2009), the total developed wall stress for each wall segment ( σ) is the sum of the scaled passive, $\sigma_{pas}$ (kPa), and active, $\sigma_{act}$ (kPa), myofiber stresses given as

$$\sigma_{i}=k_{pas,i}\sigma_{pas,i}+k_{act,i}\sigma_{act,i},$$ (8)

where $k_{pas,i}$ (kPa) and $k_{act,i}$ (kPa) are the subject-specific passive and active stress scaling factors, respectively and i=LW,RW , or SW . $\sigma_{pas,i}$ represents the developed tension without stimulation and under passive stretch.

Here, we simplified the Lumens et al. (Lumens et al., 2009) formulation of passive stress based on the exponential formula from Klotz et al. (Klotz et al., 2006) as

$$\sigma_{pas,i}={\left(\nu_{L}\left(L_{i}-L_{c,0}\right)\right)}^{\gamma },$$ (9)

where $\nu_{L}$ (μm^-1^) is a unit conversion factor, $L_{i}$ (μm) is the time-dependent sarcomere length based on myocardial strain, $L_{c,0}$ (μm) is the contractile element length at zero active stress, and γ (dimensionless) is the steepness of the length-tension relationship. To ensure our model produces an EDPVR curve with the expected exponential behavior, we employ the single-beat estimation proposed by Klotz et al. (2006) as a reference curve, which has been done previously in Krishnamurthy et al. (2013). Klotz estimated the LV EDPVR given a single-beat measurement of the end-diastolic pressure (EDP) and EDV *ex vivo* and computed approximations of the volume when the EDP is −0 and 30 mmHg. Thus, with a single end-diastolic pressure and volume point, we predict the entire EDPVR. Although the Klotz experiments were performed on LV only, we assume the RV has similar material properties to the LV, and hence, apply the same behavior to the RV. We optimize parameter γ to obtain the best fit of the model-predicted LV and RV EDPVRs to the Klotz-predicted EDPVRs using a gradient-based nonlinear least-squares optimization (Kelley, 1999).

For the active stress component, we have

$$\sigma_{act,i}=\left(\nu_{L}\left(L_{c,i}-L_{c,0}\right)\right)\left(\frac{L_{i}-L_{c,i}}{L_{se,iso}}\right)Y\left(t\right),$$ (10)

where $L_{c,i}$ (μm) is the solution to a differential equation that governs the change in sarcomere length for a given contraction velocity, $L_{i}$ (μm) is the length of the isometrically stressed series elastic element, and $L_{c,0}$ and $L_{se,iso}$ are parameters listed in Table 2. We replaced the heuristic Lumens et al. force-velocity model with a mechanical activation that has a simplified time-varying sinusoidal driving function, $Y\left(t\right)$ , simulating the contractile beating of the heart (Marquis et al., 2018) given as

$$Y_{v}\left(t\right)=\left\{\begin{array}{ll} 0.5\left(1-\cos\left(\frac{\pi t}{T_{S}}\right)\right), & 0\leq t\leq T_{S}\\ 0.5\left(1+\cos\left(\frac{\pi \left(t-T_{S}\right)}{T_{R}}\right)\right), & T_{S}\leq t\leq T_{S}+T_{R}\\ 0, & otherwise, \end{array}\right.$$ (11)

where $T_{S}=k_{TS}\;T$ (s) and $T_{R}=k_{TR}\;T$ (s) are the times of maximal systolic elastance and the end of isovolumetric relaxation, respectively, for T=60/HR (s) the period of the heart cycle at a given heart rate (HR) and $k_{TS}$ and $k_{TR}$ fractions of the cardiac cycle length (Table 2). Thus, this phenomenological formulation for chamber relaxation and contraction captures the behavior when tension develops due to myocyte contraction and collagen recruitment with physiological passive mechanics.

**TABLE 2:** Fixed model parameter values.

Name	Symbol	Unit	Value	Source
*Circulation model parameters*				
Systemic arterial viscoelastic resistance component	$R_{t,SA}$	mmHg s mL^-1^	0.08	
Pulmonary arterial viscoelastic resistance component	$R_{t,PA}$	mmHg s mL^-1^	0.02	
Mitral valve resistance	$R_{m}$	mmHg s mL^-1^	5e-4	
Tricuspid valve resistance	$R_{t}$	mmHg s mL^-1^	5e-4	
*Cardiac model parameters*				
LV wall thickness	$h_{LW}$	mm	8	(Lumens et al., 2009)
Septal wall thickness	$h_{SW}$	mm	8	(Lumens et al., 2009)
RV wall thickness	$h_{RW}$	mm	4	(Lumens et al., 2009)
Reference sarcomere length	$L_{ref}$	mm	2	(Lumens et al., 2009)
Sarcomere length at zero tension	$L_{c,0}$	mm	1.51	(Lumens et al., 2009)
Length of the isometrically stressed series elastic element	$L_{se,iso}$	mm	0.04	(Lumens et al., 2009)
Sarcomere length shortening velocity	$v_{\max}$	mm s^-1^	3.5	(Lumens et al., 2009)
*Activation function parameters*				
Maximal systole fraction	$k_{TS}$	-	0.2	
Relaxation time fraction	$k_{TR}$	-	0.2	
*Pericardium parameters*				
Pericardium shape parameter	s	-	10	(Jezek et al., 2022)
*Initial displacements*				
Axial LV midwall displacement	$x_{m,LW,0}$	cm	5	
Axial Septal midwall displacement	$x_{m,SW,0}$	cm	2	
Axial RV midwall displacement	$x_{m,RW,0}$	cm	6	
Radial midwall junction displacement	$y_{m,0}$	cm	3	

LV, left ventricle; RV, right ventricle.

#### 2.2.3 Passive stiffness and contraction parameterization

Based on EDP values for each chamber, we calculate dimesionless passive and active stress scaling factors ( $k_{pas,i}$ and $k_{act,i}$ ) based on parameters at end-diastole and end-systole, repesctively. The passive stress scaling factor relates the EDP (as in Table 1) to the TriSeg calculated chamber pressure in end-diastole such that

$$k_{pas,i}=\frac{{EDP}_{i}}{\Gamma_{i,d}\;\sigma_{pas,i,d}},$$ (12)

where $\sigma_{pas,i,d}$ is the approximated passive wall stress at end-diastole and $\Gamma_{i,d}$ is a dimensionless function of the chamber geometry including wall volume, surface area and curvature for end-diastole (Supplementary Appendix). We assume that at end-diastole $\sigma_{act,i}=0$ , then approximate $\sigma_{pas,i,d}$ using a sarcomere length of 2 μm at rest. Previous studies have shown a sarcomere length of 2.2 μm at maximal activation in canines (Rodriguez et al., 1992; Janssen, 2010), 1.9 μm in diastole in cats (de Tombe and ter Keurs, 2016); we chose 2 μm to coincide with (Lumens et al., 2009).

Similarly, the active stress scaling factor relates the ESP (as in Table 1) to the TriSeg calculated chamber pressure in end-systole such that

$$k_{act,i}=\frac{{ESP}_{i}}{\Gamma_{i,s}\sigma_{act,i,s}},$$ (13)

where $\sigma_{act,i,s}$ is the approximated active wall stress at end-systole and $\Gamma_{i,s}$ is a dimensionless function of the chamber geometry including wall volume, surface area and curvature for end-systole. At end-systole we assume 60% activation.

### 2.3 Ventricular-ventricular interactions in the TriSeg model

The three walls of the TriSeg model are mechanically coupled through a tension balance at the shared junction point where all three walls meet. The ventricular volumes are then used to calculate the chamber pressures. Given a model of wall mechanics for each segment, the axial and radial tension components are calculated at the shared junction point. All Triseg cardiac model euqations are listed in the (Supplementary Eqs SA8–SA14).

### 2.4 Pericardial constraint

We implement a pericardial constraint, $P_{peri}$ , as an external pressure onto both ventricles (i.e., the whole heart) and adapt the pericardial pressure relationship from Jezek et al. (2022) as

$$P_{peri}=\exp\left(s\left(\frac{V_{h}}{V_{h,0}}-1\right)\right),$$ (14)

where the heart volume, $V_{h}$ , is the sum of the volumes in each ventricular chamber, i.e., $V_{h}=V_{LV}+V_{RV}$ . $V_{h,0}$ is set to be 25% greater than the total end-diastolic volume of the sum of both chamber volumes, and s=10 is a shape parameter set arbitrarily to provide pericardial constraint outside normal working volume ranges.

### 2.5 Circulation model parameterization

**FIGURE 1:**
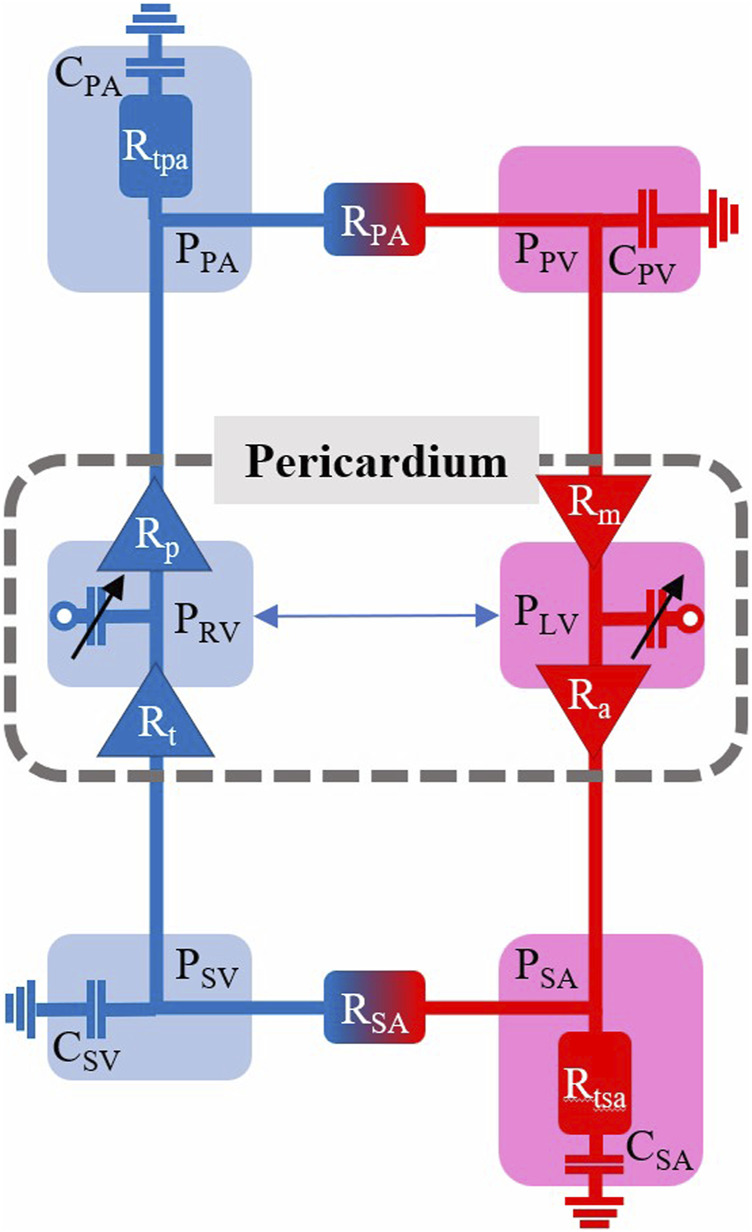
Cardiovascular model schematic with pressures (P), resistances (R), and compliances (C) indicated for the six model compartments with a pericardium encapsulating the left and right ventricles (LV, RV). SA—systemic arterial. PA—pulmonary arterial. SV—systemic venous. PV–pulmonary venous.

Figure 1 shows a schematic of the circulation model comprising six compartments. We determine vascular model parameters using two strategies: 1) scaling compartmental volumes as percentages of total blood volume and approximations of blood distribution, and 2) scaling compartmental pressures based on SBP and arterial diastolic blood pressures (DBP). Systemic and pulmonary compliances were approximated as the ratio between the maximal stressed volume and maximal pressure generated in each compartment; the unstressed volume for each compartment is defined based on Beneken (1979), and the ratio of stressed to unstressed volume is based on Beneken (1979). Systemic and pulmonary vascular resistances were set as the ratio between the change in pressure between compartments based on Boron and Boulpaep (2016) and the overall cardiac output. Further details are given in Supplementary Appendix. All cardiovascular parameters for the healthy case are listed in Table 2.

### 2.6 Healthy and dysfunction simulations

The healthy (H) case is defined based on normative hemodynamic data including LV and RV pressures and volumes (Table 1) and cardiac and circulation model parameter values (Table 2). To simulate acute SD, we reduce overall active myofiber stress by scaling $k_{act,i}$ to 60% (moderate, M) and 40% (severe, S) of the nominal value. Similarly, for acute DD, we increase passive stiffness, as done in previous studies (Kadry et al., 2020; Comunale et al., 2021), by scaling $k_{pas,i}$ by a factor of 7 (M) and 15 (S). For both SD and DD, the septum is considered part of the LV. We assume the mechanisms of RV dysfunctions are the same as those for the LV (Comunale et al., 2021). For the dysfunction simulations, we increase the ratio of stressed to unstressed blood volume to 30% in the systemic and pulmonary compartments as described in Jones et al. (2021). All simulations shown here are at a HR of 60 bpm, and the Supplemental Material S1 provides select results for HR at 80 and 100 bpm. Redistributed blood volume ratios are listed in Table 3. We run the model for 20 heartbeats which is sufficient to equilibrate transients, and then solve for two more beats for all model results.

**TABLE 3:** Compartment volume fractions adapted from Beneken (1979) and pressures (Boron and Boulpaep, 2016) for the cardiovascular model.

Compartment	Symbol	TBV fraction	Unstressed volume fraction		Maximal pressure ( ${\hat{P}}_{M}$ )	Mean pressure ( ${\hat{P}}_{bar}$ )	Minimal pressure ( ${\hat{P}}_{m}$ )
			Healthy Case	Disease Case			
					mmHg	mmHg	mmHg
Left ventricle	LV	0.03	-	-	121	-	4
Systemic arteries	SA	0.2	0.70	0.60	120	95	80
Systemic veins	SV	0.54	0.90	0.8	6	4	4
Right ventricle	RV	0.03	-	-	26	-	4
Pulmonary arteries	PA	0.05	0.40	0.35	25	15	10
Pulmonary veins	PV	0.15	0.90	0.75	8	5	2

TBV, total blood volume.

### 2.7 Compensatory volume loading

As a VVI challenge, we increased circulating blood volume from 100% to 350%. In this study, we volume loaded to achieve a mean systemic arterial pressure ( ${\bar{P}}_{sa}$ ) of ∼95 mmHg that represents the fully compensated state as a baseline point for comparison between the healthy and disease cases. No other changes (e.g., increasing systemic or pulmonary arterial resistance) were performed, and all cardiovascular results are emergent properties of this modeling approach. Note that the ability to achieve compensation to this baseline point was limited in some of the severe HF simulations. Therefore, these “baseline severe” points may be unattainable physiologically, though their simulations provide useful insights.

## 3 Results

### 3.1 Baseline model calibration

**FIGURE 2:**
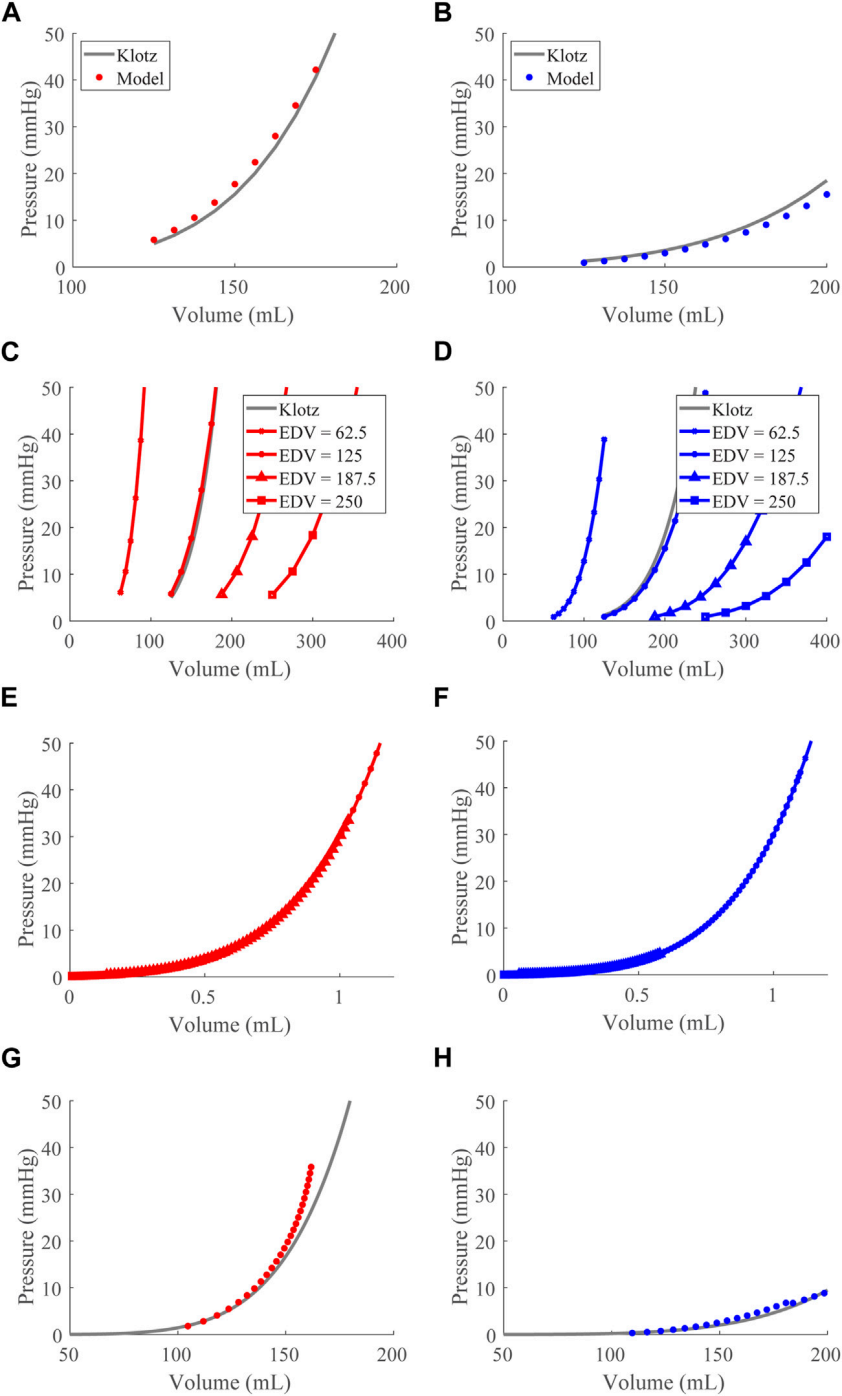
End-diastolic pressure-volume relationship (EDPVR) optimization and validation. **(A)** and **(B)**. The left (red) and right (blue) ventricular model-predicted *ex vivo* EDPVRs (circles) plotted with the Klotz EDPVRs (solid black curve) for the optimized γ value. **(C)** and **(D)**. Reproduction of the Klotz experiment (2006) (Section 3.1). The *ex vivo* EDPVR across various heart sizes with the Klotz EDPVR curve at a volume of 125 mL (solid grey). **(E)** and **(F)**. The EDPVR curves from **(C)** and **(D)** with the volumes normalized according to Klotz et al. (2006). The normalized curves lay on top of each other. **(G)** and **(H)**. The left (red) and right (blue) *in vivo* model EDPVRs (points) plotted with the Klotz EDPVRs (curves).

To ensure the model produced the appropriate EDPVR while volume loaded, we used the Klotz approximated EDPVR curve as a reference and optimized γ (Figure 2). The model predicted *ex vivo* EDPVRs for the LV (Figure 2A) and RV (Figure 2B) (i.e., no pericardium and isolated from the systemic and pulmonary circulations) are plotted with the associated Klotz EDPVR showing agreement when γ=7.5 . We further validate the model predictions by simulating experiments performed by Klotz et al., wherein LV EDPVRs from several human hearts were shown to have the same shape after normalizing the EDV (2006). In these simulations, we used EDV=62.5,125,187.5 , and 250 mL in both the RV and LV to represent different heart sizes (Figures 2C,D) and achieved identical normalized EDPVRs for both ventricles (Figures 2E,F). We then used these *ex vivo*-validated EDPVR behaviors in the full model (*in vivo*; Figures 2G,H), demonstrating the influence of pericardial constraint at high volumes for the LV in particular.

**FIGURE 3:**
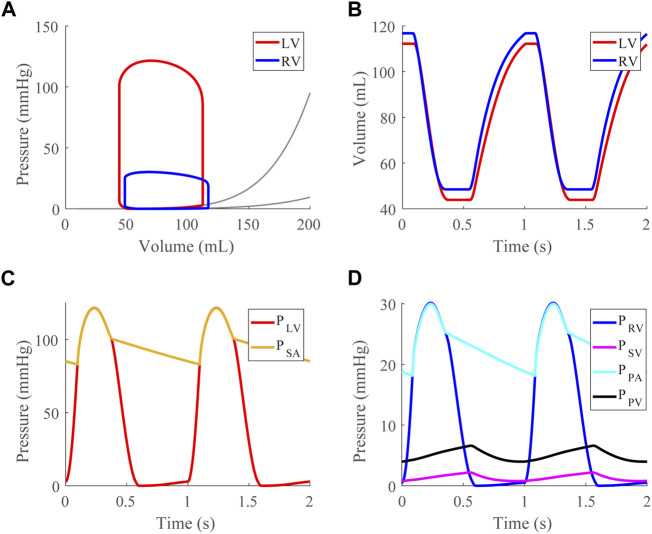
Healthy case at a mean systemic arterial, SA, pressure of 95 mmHg (baseline). **(A)**. Left (LV) and right (RV) ventricular pressure-volume loops shown with approximated EDPVR curves (Klotz et al., 2006). **(B)**. LV and RV volume time courses. **(C)**. LV and SA pressure time courses. **(D)**. RV, systemic venous (SV), pulmonary arterial (PA), and pulmonary venous (PV) pressure time courses.

**FIGURE 4:**
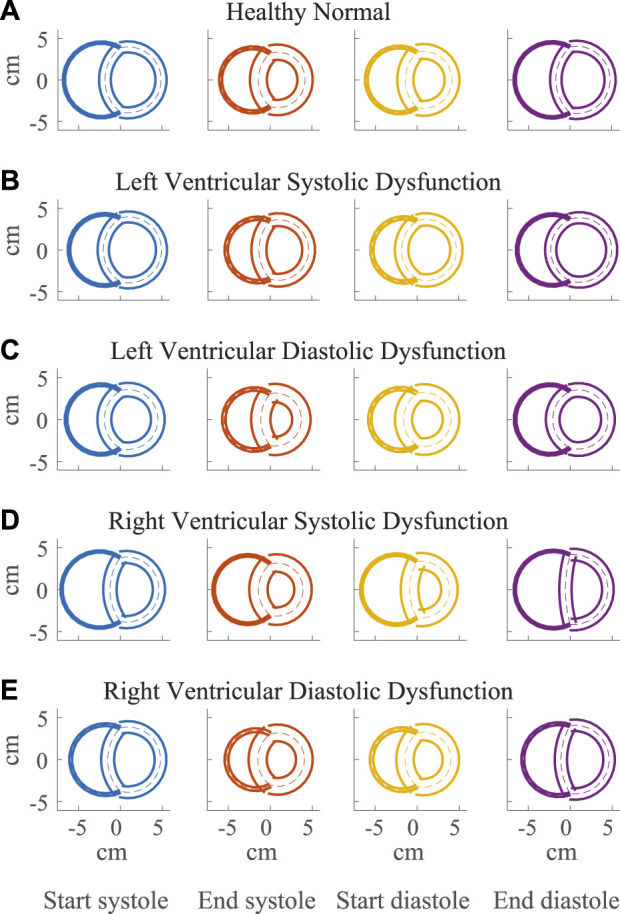
Visualization of simulated ventricular geometry during the cardiac cycle in the healthy **(A)** and severe dysfunction **(B–E)** cases. The geometry of the Triseg model, representative of a biventricular transverse plane view, is illustrated at several times in the cardiac cycle, including start and end of systole and diastole with left ventricle (LV) on the left and right ventricle (RV) on the right. The overlap of the ventricular walls and septum follows from the simplifying assumption of the TriSeg model geometry. Dashed lines represent the TriSeg midwall, creating intersection points with balanced tensions. At end-diastole, LV and RV lumens are maximally full. During contraction, the walls progressively thicken until end-systole. In RV dysfunction, the effect of elevated RV preload on septal curvature is apparent at end-diastole, where the septum has become much more flattened compared to the other cases.

After validating the passive properties of the cardiac model, we simulated the healthy case at a baseline TBV of 4.6 L, generating pressure-volume loops (Figure 3A), time courses for the LV and RV volumes (Figure 3B), and compartmental pressures (Figures 3C,D). $P_{sa}$ is 120/80 mmHg with a mean of ∼95 mmHg. Figure 4 A shows ventricular geometry during VVI at several points in the cardiac cycle, displaying fluctuations in the LW, RW, and SW. The LW volume increases ∼60% between end-diastole and end-systole, and the RW volume increases ∼70%. Table 3 lists functional healthy case metrics.

### 3.2 Dysfunction cases

**FIGURE 5:**
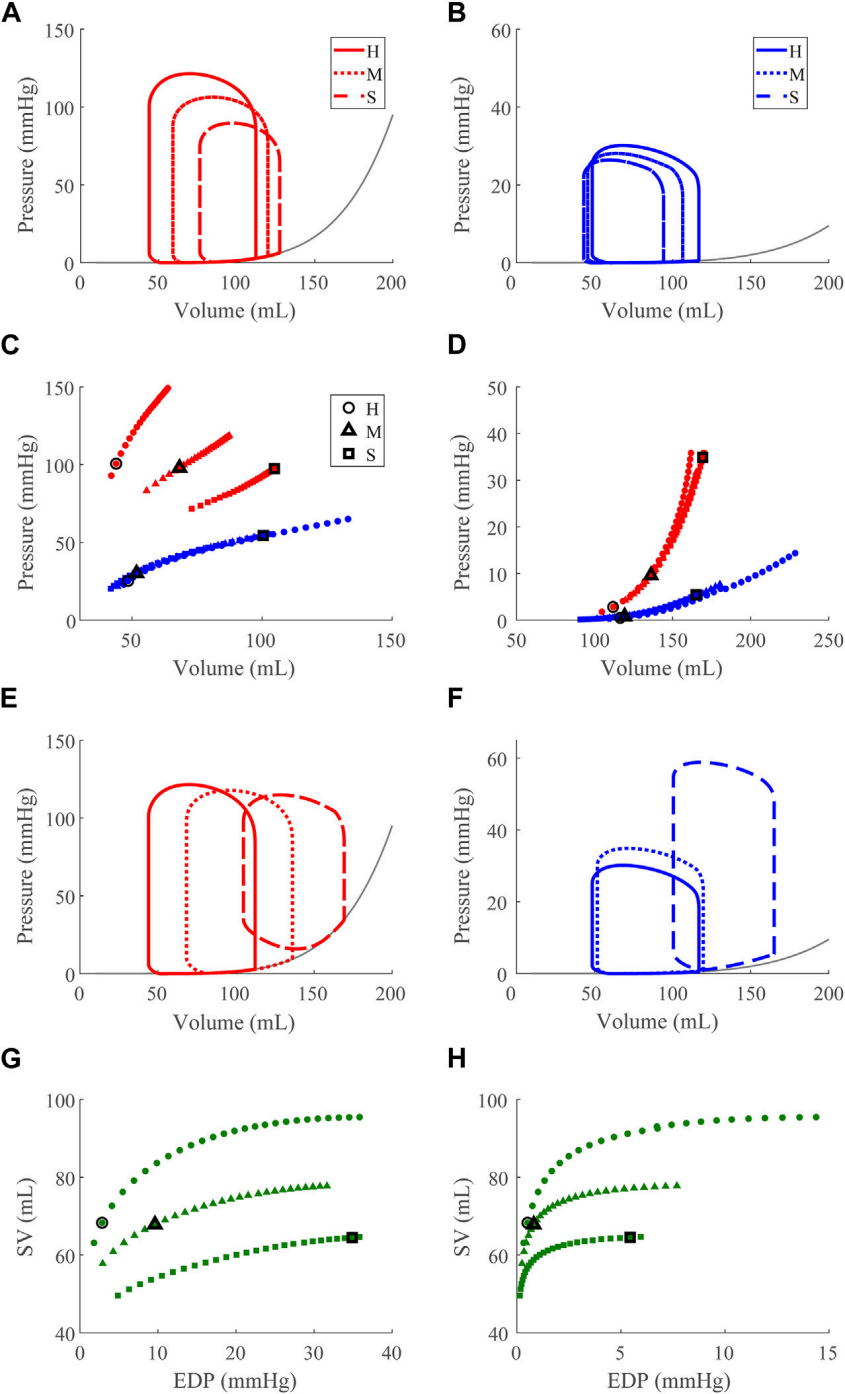
Left ventricular (LV) systolic dysfunction (SD) simulation. From the healthy (H) case, LV contractility is reduced (Section 2.5) for the moderate (M) and severe (S) cases. **(A, B)** The left (red) and right (RV, blue) ventricular pressure-volume loops at the healthy total blood volume for the H, M, and S cases shown with the healthy-case Klotz EDPVR curve (gray). **(C, D)**. The end-systolic pressure-volume relationship and the end-diastolic pressure-volume relationship for the LV and RV. After volume loading, the baseline at full compensation is denoted by the black bolded marker. **(E, F)**. The LV and RV pressure-volume at full compensation, corresponding to the markers in C and D. **(G, H)**. The LV and RV Frank-Starling relationship curve for the H, M, and S case with baseline indicated by the black bolded marker.

**FIGURE 6:**
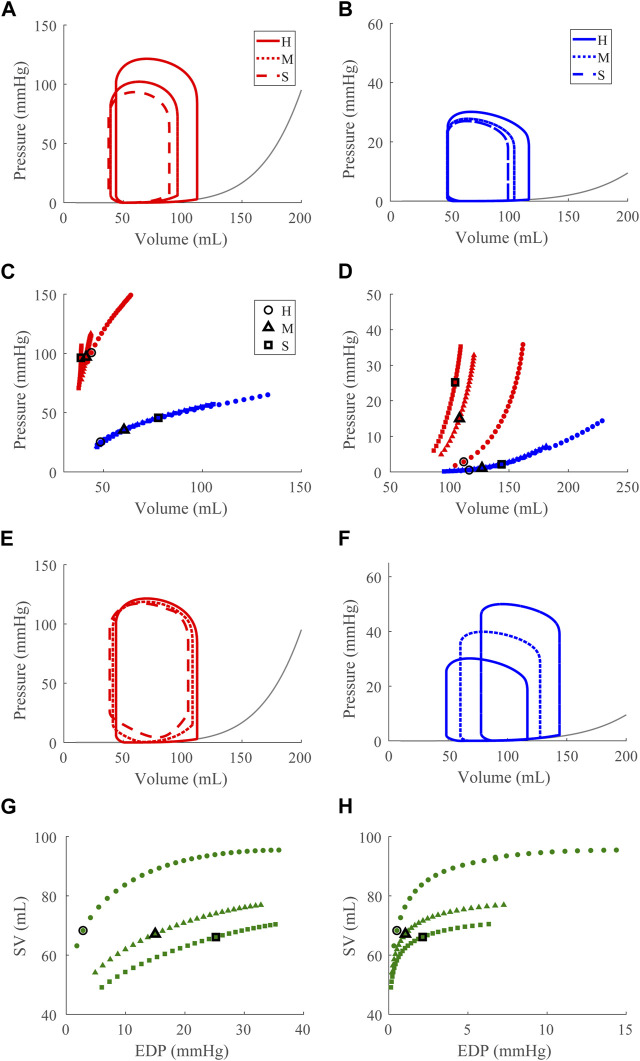
Left ventricular (LV) diastolic dysfunction (DD) simulation. From the healthy (H) case, the LV passive stiffness is increased (Section 2.5) for the moderate (M) and severe (S) cases. **(A, B)**. The left (red) and right (RV, blue) ventricular pressure-volume loops at the healthy total blood volume for the H, M, and S cases shown with the healthy-case Klotz EDPVR curve (gray). **(C, D)**. The end-systolic pressure-volume relationship and the end-diastolic pressure-volume relationship for the LV and RV. After volume loading, the baseline at full compensation is denoted by the black bolded marker. **(E, F)**. The LV and RV pressure-volume loops at full compensation, corresponding to the markers in C and D. **(G, H)**. The LV and RV Frank-Starling relationship curve for the H, M, and S case with baseline indicated by the black bolded marker.

**FIGURE 7:**
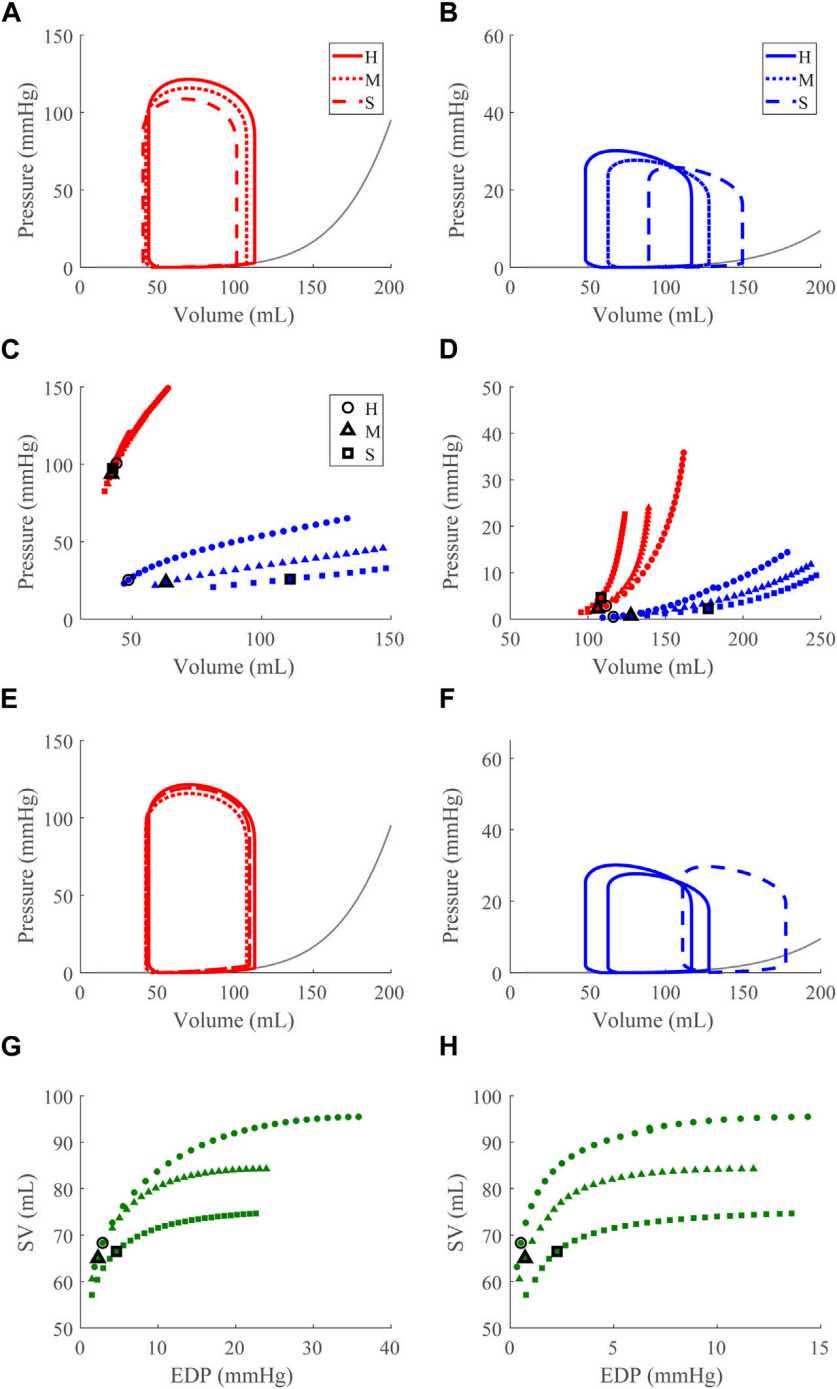
Right ventricular (RV) systolic dysfunction (SD) simulation. From the healthy (H) case, the RV contractility is reduced (Section 2.5) for the moderate (M) and severe (S) cases. **(A, B)**. The left (red) and right (RV, blue) ventricular pressure-volume loops at the healthy total blood volume for the H, M, and S cases shown with the healthy-case Klotz EDPVR curve (gray). **(C, D)**. The end-systolic pressure-volume relationship and the end-diastolic pressure-volume relationship for the LV and RV. After volume loading, the baseline at full compensation is denoted by the black bolded marker. **(E, F)**. The LV and RV pressure-volume loops at full compensation, corresponding to the markers in C and D. **(G, H)**. The LV and RV Frank-Starling relationship curve for the H, M, and S case with baseline indicated by the black bolded marker.

**FIGURE 8:**
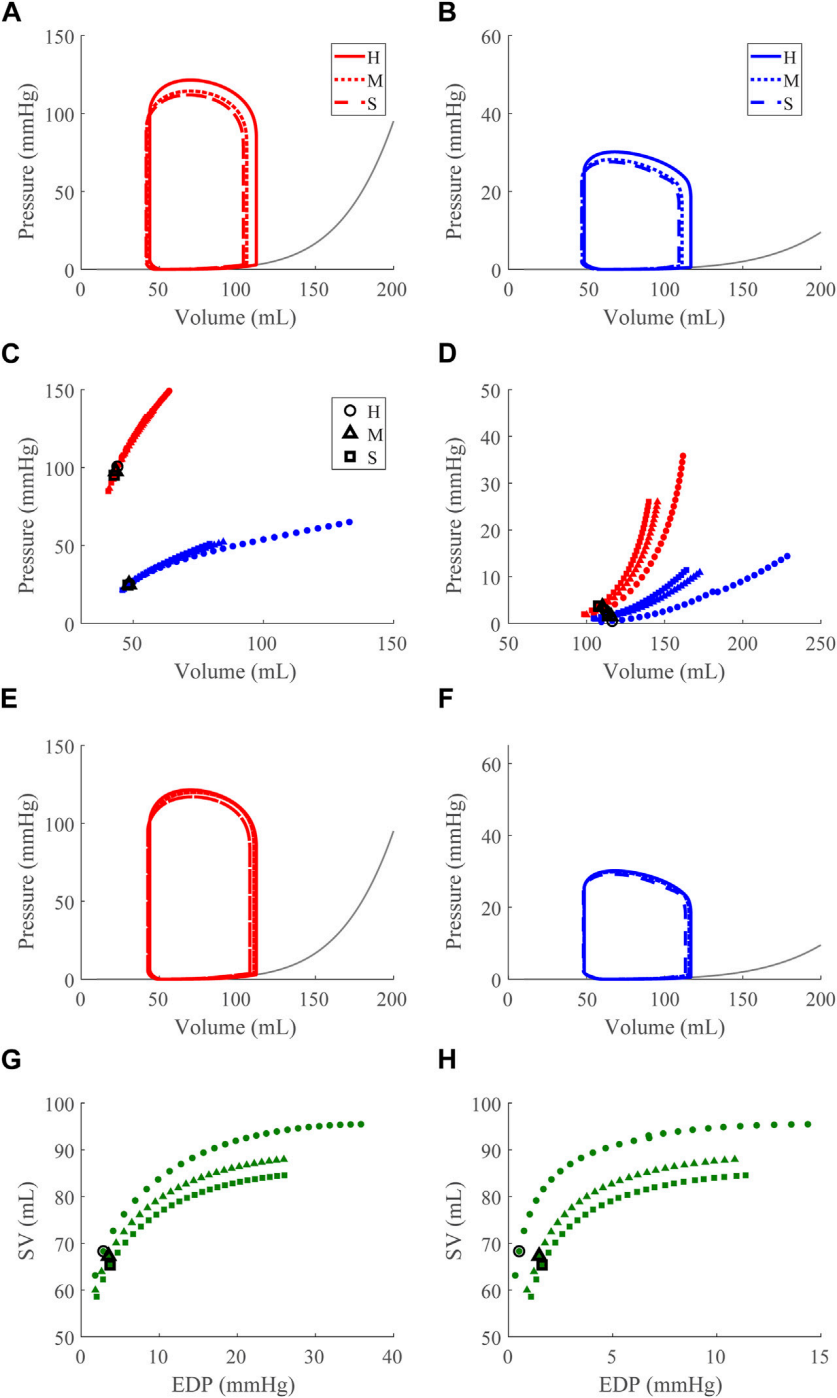
Right ventricular (RV) diastolic dysfunction (DD) simulation. From the healthy (H) case, the RV passive stiffness is increased (Section 2.5) for the moderate (H) and severe (S) cases. **(A, B)**. The left (red) and right (RV, blue) ventricular pressure-volume loops at the healthy total blood volume for the H, M, and S cases shown with the Klotz EDPVR curve (gray). **(C, D)**. The end-systolic pressure-volume relationship and the end-diastolic pressure-volume relationship for the LV and RV. After volume loading, the baseline at full compensation is denoted by the black bolded marker. **(E, F)**. The LV and RV pressure-volume loops at full compensation, corresponding to the markers in C and D. **(G, H)**. The LV and RV Frank-Starling relationship curve for the H, M, and S case with baseline indicated by the black bolded marker.

**FIGURE 9:**
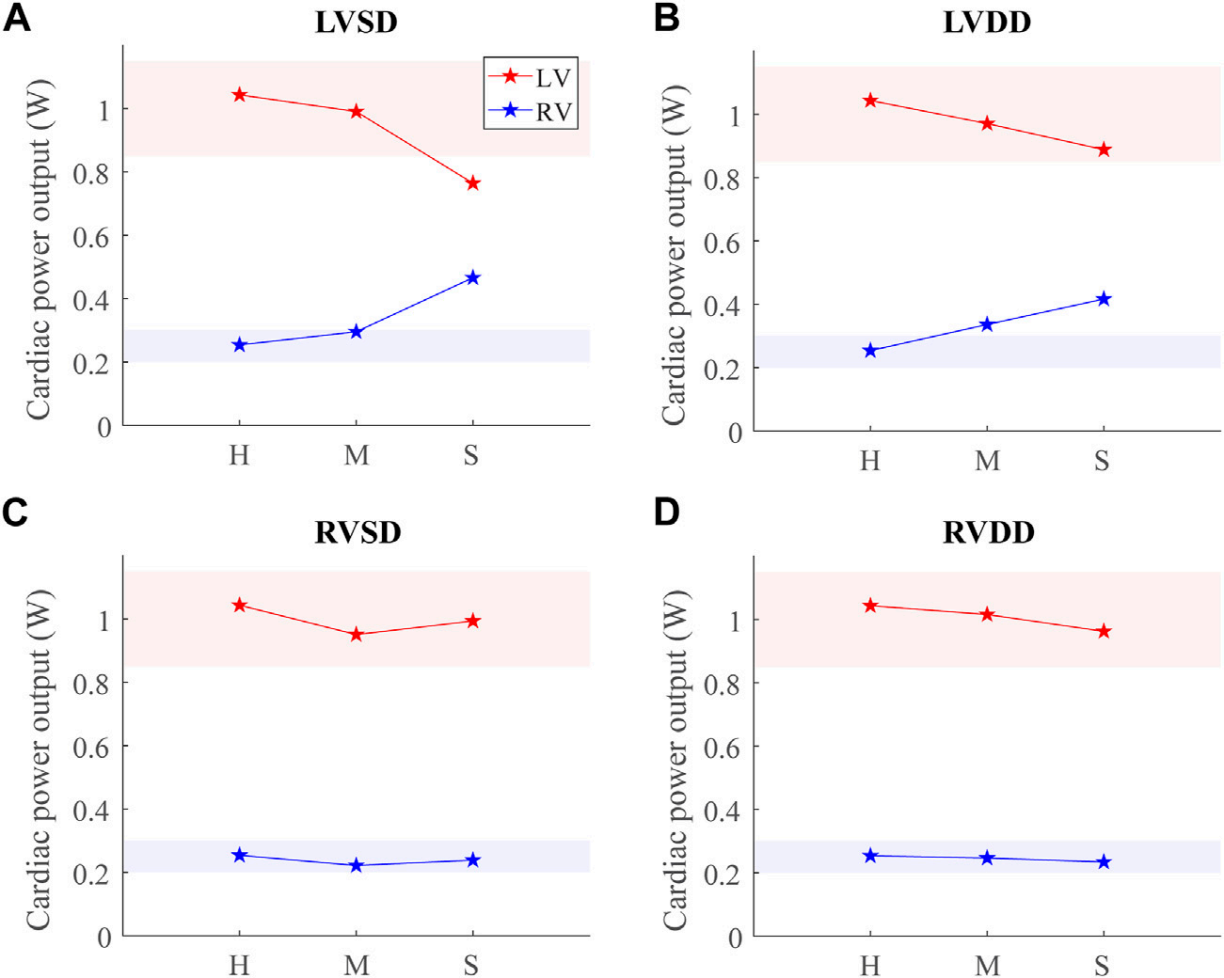
Cardiac power output (CPO) at baseline for healthy (H), moderate (M), and severe (S) cases for the four dysfunction simulations. Healthy-normal ranges for the left ventricular (LV, red) and right ventricular (RV, blue) CPO are indicated by the shaded regions. **(A)** LV systolic dysfunction (LVSD). **(B)** LV diastolic dysfunction (LVDD). **(C)** RV systolic dysfunction (RVSD). **(D)** RV diastolic dysfunction (RVDD).

**FIGURE 10:**
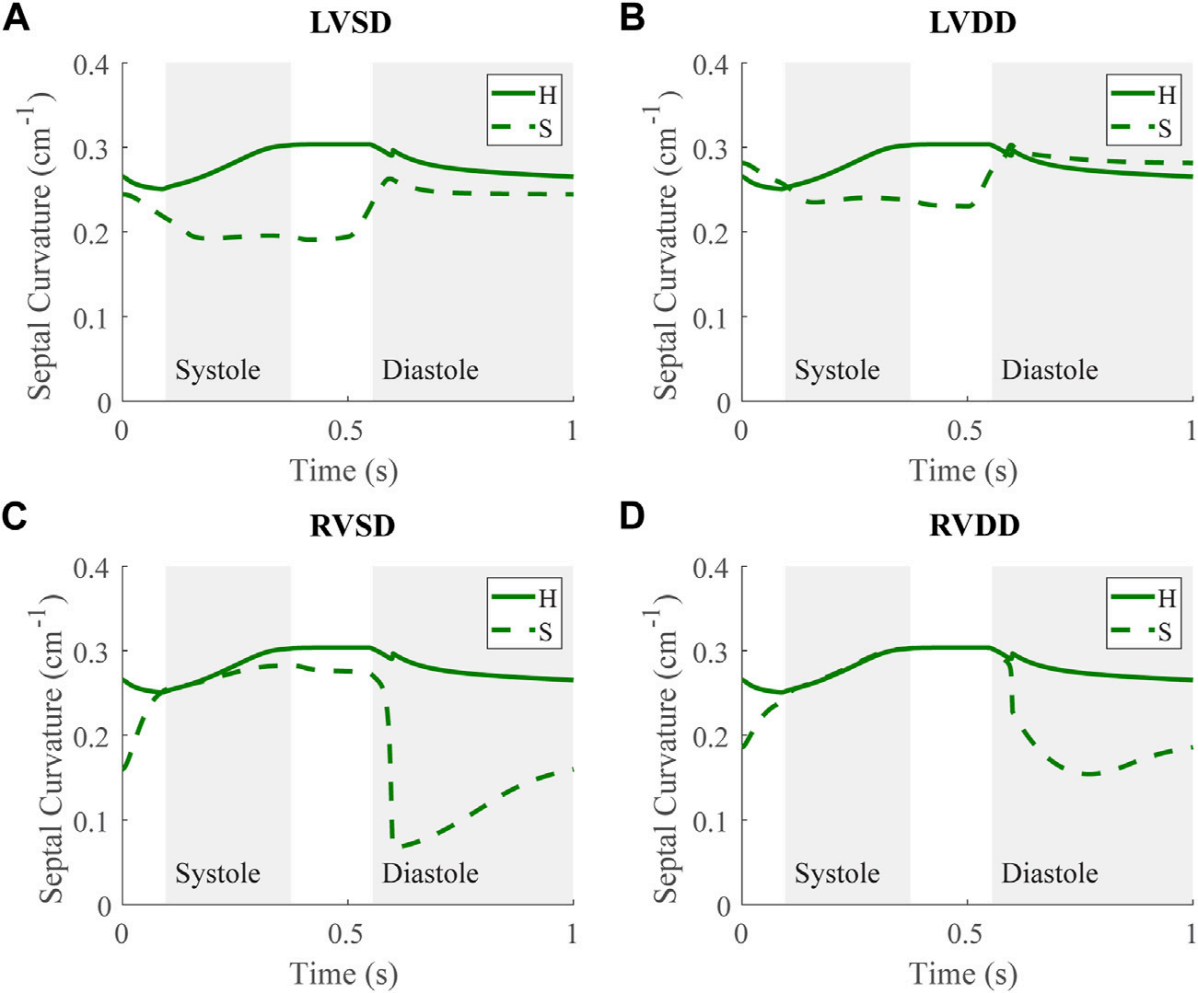
Septal curvature time courses at baseline for the severe (S) case for the four dysfunction simulations with curvature at baseline for the healthy (H) case. Systole and diastole for the H case are indicated by the shaded regions. Positive curvature indicates bowing of the septum toward the right ventricle (RV) and a negative curvature would indicate bowing toward the left ventricle (LV). **(A)** LV systolic dysfunction (LVSD). **(B)** LV diastolic dysfunction (LVDD) **(C)** RV systolic dysfunction (RVSD). **(D)** RV diastolic dysfunction (RVDD).

Compensatory volume loading simulations were performed for the healthy (H) and dysfunction cases, including LVSD, LVDD, RVSD, and RVDD (Figures 4–8) with two degrees of severity, moderate (M) and severe (S). Figures 4B–E visualizes the ventricular geometry at several points in the cardiac cycle for the severe dysfunction cases only. For each dysfunction (and each Figure), we show the pressure-volume loops in panels **A** (LV) and **B** (RV) at a normal circulating blood volume. Panels **C** and **D** show end-systolic pressure volume (ESPVR) and EDPVR curves, respectively, during volume loading. The ESPVRs show physiological nonlinearity (Burkhoff, Mirsky, and Suga, 2005). The bold black marker in panels **C** and **D** indicates the baseline point with fully compensated circulating blood volume to maintain ${\bar{P}}_{SA}=95$ mmHg and SV, and panels **E** and **F** show pressure-volume loops at this baseline point. The Frank-Starling curves for the LV and RV are shown in panels **G** and **H**, respectively. To compare ventricular pumping power across conditions, we compute CPO (Figure 9) at the baseline points in Figures 5–8 **E and F**. Septal curvature as a function of time in the healthy and dysfunction cases are provided in Figure 10. Finally, Table 4 lists functional metrics for the healthy, moderate, and severe cases for LVSD, LVDD, RVSD, and RVDD.

**TABLE 4:** Model predictions for several cardiac metrics for healthy and all dysfunction cases.

		SV	EF	CO	CPO	ESP	EDP	ESV	EDV
		(mL)	(%)	(mL min^-1^)	(W)	(mL)	(mL)	(mL)	(mL)
*Healthy*									
	LV	68	61	4.1	1.0	100	2.8	44	112
	RV	68	59	4.1	0.25	25	0.51	48	116
*LV Systolic Dysfunction*									
Moderate	LV	68	50	4.1	0.99	98	9.6	68	136
	RV	68	57	4.1	0.30	30	0.81	52	119
Severe	LV	64	38	3.8	0.76	98	35	105	169
	RV	64	39	3.8	0.47	54	5.4	100	165
*LV Diastolic Dysfunction*									
Moderate	LV	67	62	4.0	0.97	97	15	41	108
	RV	67	53	4.0	0.34	35	1.1	60	127
Severe	LV	66	63	4.0	0.89	96	25	39	105
	RV	66	46	4.0	0.42	46	2.1	78	144
*RV Systolic Dysfunction*									
Moderate	LV	65	61	4.9	0.95	94	2.2	42	106
	RV	65	51	4.9	0.22	24	0.72	63	128
Severe	LV	66	61	3.9	0.99	97	4.6	42	108
	RV	66	37	3.9	0.24	26	2.3	111	178
*RV Diastolic Dysfunction*									
Moderate	LV	67	61	4.0	1.01	98	3.5	43	110
	RV	67	58	4.0	0.25	25	1.5	48	116
Severe	LV	65	61	3.9	0.96	95	3.7	43	107
	RV	65	58	3.9	0.23	25	1.6	48	113

SV, Stroke Volume; EF, Ejection Fraction; CO, Cardiac Output; CPO, Cardiac Power Output; ESP, End Systolic Pressure; EDP, End Diastolic Pressure; ESV, End Systolic Volume; EDV, End Diastolic Volume. LV, Left Ventricle; RV, Right Ventricle.

#### 3.2.1 Left ventricle systolic dysfunction

Reducing $k_{act,LW}$ and $k_{act,SW}$ to simulate acute LV SD reduces LV ESP, increases ESV, and increases EDP along the EDPVR curve (Figure 5A). Whereas RV ESP decreases as in the LV, although to a lesser degree, and RV ESV and EDV decrease (Figure 5B). The areas of the LV and RV pressure-volume loops decrease with dysfunction, indicating decreased CPO. Under volume loading conditions, the impact of the impaired pumping is evident in the decreased slope of the LV ESPVR (Figure 5C); LV and RV EDPVR are unaffected (Figures 5C,D). At full compensation, the ESV increases [40% (M) and 90% (S)] and filling pressure increases [125% (M) and 520% (S)] in both ventricles (Figures 5E,F). As a result, LV pressure-volume area decreases [3% (M) and 8% (S)] and is met by a compensatory increase in RV pressure-volume area [1% (M) and 10% (S)]. The RV EDV increases significantly from the healthy to severe case, and physiologically full compensation may be limited by constraint of the pericardium. As expected, with impaired LV contractility the Frank-Starling mechanism decreases and greater EDPs are needed to maintain SV (Figure 5G). The impaired LV also results in a decrease in the RV Frank-Starling mechanism (Figure 5H).

#### 3.2.2 Left ventricular diastolic dysfunction

Increasing $k_{pas,LW}$ and $k_{pas,SW}$ to simulate acute LV DD reduces EDV and reduces ESP (Figure 6A). As in the LV, the RV EDV and ESP decrease, but the RV EDP decreases with EDV, following the RV EDPVR (Figure 6B). Under volume loading conditions, increased LV contractility to maintain SV (Aurigemma, Zile, and Gaasch, 2006; Rommel et al., 2018) is observed by the increased slope of the LV ESPVR (Figure 6C); the imposed stiffening of the LW is evident in the upward, leftward shift and increased slope of the LV EDPVR (Figure 6D). RV systolic and diastolic function is maintained. At full compensation, LV EDV and ESP partially normalize (Figure 6E). LV EDP increases substantially from the healthy case (865% (M) and 1450% (S)). The LV pressure-volume area decreases (20% (S)) and is met by a compensatory increase in RV pressure-volume area (40% (S)) as the ESP and EDV increase with greater dysfunction (Figure 6F) that results from the Frank-Starling mechanism. As expected, the Frank-Starling behavior is depressed, and greater filling pressures are needed to achieve the necessary preload in the stiffer LV myocardium to maintain SV (Figure 6G). The RV Frank-Starling mechanism is likewise affected by the impaired LV (Figure 6H)

#### 3.2.3 Right ventricular systolic dysfunction

Decreasing $k_{act,RW}$ to simulate acute RV SD leads to a small reduction in LV ESP and EDV (Figure 7A). For the RV, there are slight decreases in RV ESP and increases in EDV (Figure 7B). The areas of the LV and RV pressure-volume loops decrease slightly with dysfunction. Under volume loading conditions, impaired pumping is evident in the decreased slope of the RV ESPVR curve (Figure 7C) while the LV ESPVR is unaffected. However, with dysfunction and at high volumes, the LV EDPVR slope increases, indicating increased stiffness of the LV (Figure 7D). Since there are no changes to the intrinsic LV free wall mechanics, this is an *apparent stiffening* due to mechanical interactions between the ventricles. The slope of the RV EDPVR curve decreases slightly with dysfunction as the flattening of the septum allows for more RV filling given the same pressure. At full compensation, the LV pressure-volume loop is mostly maintained from healthy (Figure 7E) and the RV systolic pressures are restored with increased EDV (16% (M) and 50% (S), Figure 7F). As expected, the Frank-Starling mechanism is depressed and greater EDP pressures are required to maintain SV as dysfunction increases (Figures 7G,H).

#### 3.2.4 Right ventricular diastolic dysfunction

Increasing $k_{pas,RW}$ to simulate acute RV DD slightly reduces EDP and ESP for both the LV and RV (Figures 8A,B). Under volume loading conditions, the systolic behavior is maintained for both ventricles (Figure 8C); the slope of the EDPVR curve increases for both ventricles indicating increased stiffness (Figure 8D). For the RV, this increased stiffness reflects our imposed stiffening of the RV wall; for the LV, this *apparent stiffening* is not due to any intrinsic changes to LV wall mechanics and instead arises from interactions between the ventricles. At full compensation, the pressure-volumes loops are mostly restored for both ventricles (Figures 8E,F). Again, the Frank-Starling mechanism is depressed as expected (Figures 8G,H).

### 3.3 Cardiac power output

Comparing CPO across all conditions at the baseline point demonstrates an inverse relationship between the LV and RV CPO, such that when the LV CPO decreases, RV CPO increases (Figures 9A,B). This finding suggests RV compensation is required to maintain pressure and flow when LV function (systolic or diastolic) is impaired. The changes in LV CPO for RV dysfunction are negligible (Figures 9C,D). Interestingly, despite a 15-fold increase in $k_{pas}$ to simulate severe DD, neither LV CPO nor RV CPO decreased below a nominally normal range (shaded regions) (Fincke et al., 2004; Yildiz and Yenigun, 2021). Only in conditions of severe LV dysfunction did RV CPO increase above the normal range (Figures 9A,B). Figure 9 displays results at 60 bpm, and the Supplemental Material S1 shows LV and RV CPO at 80 bpm and 100 bpm. At higher HR, the baseline point occurs at greater circulating blood volume (results not shown). LVSD shows a substantial HR dependence, decreasing more rapidly as HR increases (Supplementary Figure S1). LVDD shows a slight HR dependence, whereas the other dysfunction cases do not.

### 3.4 Septal flattening

Along with spatial representations of the LV and RV geometries (Figure 4), the TriSeg model allows analysis of SW curvature over time due to force balances at the SW insertion points and pressure balances along the wall (Figure 10). In all healthy and disease cases tested here, curvature is always positive, indicating bowing into the RV, but differences in curvature over time are evident with disease. In the healthy case, the SW deflects towards the RV throughout the cardiac cycle, and wall thickness increases from end-diastole to end-systole (Figure 4A). Septal curvature decreases somewhat, indicating mild flattening of the septum at the start of systole, and increases to a maximal curvature just before the aortic valve closes (Figure 10). During isovolumic relaxation, the curvature is relatively constant at about 0.30 cm^−1^, and during diastole curvature decreases, reaching a minimum of about 0.25 cm^−1^. For severe LVSD, septal flattening can be visualized during systole both spatially (Figure 4B) and temporally (Figure 10A) with near normal curvature during diastole. For severe LVDD, some flattening occurs during systole (Figure 4C), and greater-than-normal curvature occurs during diastole (Figure 10B). This increased bowing of the septum toward the RV during diastole helps compensate for the impaired LV by increasing the LV chamber volume. In contrast, for both the severe RVSD and RVDD cases, septal dynamics deviate the most from healthy during diastole (Figures 4D,E, 10C,D) with again more dramatic changes in SD compared to DD. For RVSD, dramatic RV dilation causes the SW flattening; for RVDD, limited RV dilation (due to stiffening) leads to SW flattening, which allows for maintained RV filling volumes.

## 4 Discussion

Using a modified TriSeg heart model developed here and the closed-loop, lumped-parameter circulation model, our results highlight the interdependence of the ventricles and demonstrate the effects of LV failure on RV function and *vice versa*. Specifically, our simulations reveal expected RV compensation for the failing LV and unexpected apparent LV DD in the presence of RV SD and RV DD, which may contribute to the HFpEF phenotype. Moreover, our simplified parameterization framework of the TriSeg model serves as a foundation for subject-specific investigations.

### 4.1 Model performance in the healthy and dysfunction cases

For the healthy case, the model-simulated LV and RV pressure-volume relationships, Frank-Starling curves, and septal curvature analyses demonstrate physiological cardiac mechanics for a typical 70 kg human (Figure 3). In particular, the EDPVR, ESPVR, and Frank-Starling curves generated have the expected shapes. Using the Klotz et al. (2006), we calibrated the EDPs as done previously (Krishnamurthy et al., 2013), and our model EDPVR behavior shows satisfactory congruence with experiment results, capturing EDPVR normalization (Klotz et al., 2006). Thus, we are confident the changes produced by the model EDPVR in response to model interventions appropriately reflect physiological behaviors.

Our model also exhibits the expected pathologies associated with LV SD and LV DD. The LVSD simulations (Figure 5) demonstrate reduced ejection fraction, increased LV systolic and diastolic volumes, increased EDP, reduced end-systolic elastance, and a flattened ESPVR with a maintained EDPVR as found clinically (Aurigemma, Zile, and Gaasch, 2006). RV systolic function is maintained with LVSD as indicated by the unchanged ESPVR curve (Figure 5C), but the RV ejection fraction decreases with increasing LV systolic impairment as the RV dilates (Table 3; Figure 5F). Our LVDD simulations (Figure 6) represent the most restrictive DD case and reproduce the expected behaviors: maintained ejection fraction, increased EDPs, decreased EDVs, a leftward, upward shift of the EDPVR curve, and a maintained or increased end-systolic elastance, consistent with the clinical literature (Aurigemma, Zile, and Gaasch, 2006). Interestingly, in LVDD the end-systolic elastance, as measured by the ESPVR, increased (Figure 6C). This response has been reported clinically to varying degrees (Rommel et al., 2018). Enhanced contractility may be a response to increasing preload from impaired LV filling. Hence, model agreement with previously reported behaviors gives confidence in our model predictions.

### 4.2 Ventricular-ventricular interactions in HFrEF

Our simulations reveal increased RV work in response to attenuated LV pump function in acute LVSD. In this case, LV myofiber contraction is intrinsically impaired, so greater LV preloads are necessary to generate a given pressure. As a result, the RV has a corresponding increase in CPO to generate the increased pressure required to supply sufficient preload for LV contraction via the Frank-Starling mechanism. Chronically elevated RV CPO in LVSD can eventually lead to deleterious remodeling of the RV. Indeed, elevated RV CPO has been independently correlated to poor outcomes in HFrEF (Yildiz and Yenigun, 2021). Interestingly, LVSD simulated at several HRs shows a frequency dependence where higher HRs result in progressively worse LV function (decreased CPO) and greater RV compensation (Supplementary Figure S1).

### 4.3 Apparent LV stiffening due to ventricular-ventricular interactions

Apparent LV stiffening arises, as shown by the increased steepness of the LV EDPVR curve (Figures 7D, 8D) in our simulations of RVSD and to a lesser degree RVDD, despite no changes to intrinsic properties of the LV free wall or septum as in the LV case. At baseline the effects on the LV are minimal (Figures 7E, 8E), and increased HR does not result in substantial changes in LV or RV CPO (Supplementary Figure S1). However, as preload increases, deviations from the healthy case become more evident. Our modeling approach supports that this resulting LV DD is due to mechanical interactions between the LV and the failing RV. Note, if the LV and RV were independent bodies connected in series by the circulations, increases in circulating blood volume (to increase RV preload), would increase the LV end diastolic pressure and volume, but not the shape of the LV EDPVR. To assess the pericardium’s role, we repeated the severe RVSD and RVDD cases in its absence. Though we observed a slight contribution to the increased steepness of the LV EDPVR, particularly at full compensation (results not shown), the pericardium does not substantially impact LV EDPVR steepness. In contrast, septal dynamics appear to play a significant role. With RVSD and RVDD, we observed septal flattening temporally (Figures 10C,D) and spatially (Figures 4D,E), which effectively reduces LV volume and may impair contractile dynamics given the non-cylindrical shape. Hence, LV DD in the RV dysfunction cases is most likely a result of VVI mediated by septal dynamics.

### 4.4 Ventricular-ventricular interactions in classic HFpEF

Our simulation results reveal three dysfunction cases that correspond to HFpEF. Classically, HFpEF is characterized by preserved LV ejection fraction and impaired LV filling as the common phenotype (Lekavich et al., 2015), which are present in the LVDD, RVSD, and RVDD cases. In the LVDD case, we impose LV stiffening which directly impairs LV filling. The attenuated LV function and corresponding augmented RV function is reflected in the increased RV CPO. In LVDD, the stiffer LV myocardium requires greater pressures to generate the preload needed to maintain SV and systemic arterial pressure, provided by the increased pump function of the RV. In our RV dysfunction simulations, LV function is mostly maintained at volumes required to maintain a mean arterial blood pressure of ∼95 mmHg (Figures 7E, 8E). Here, LV DD occurs at greater circulating blood volume, resulting in higher preloads (Figures 7D, 8D) that correspond to exercise (Fudim, Sobotka, and Dunlap, 2021) or volume overload (Miller, 2016).

The LV DD present at high preloads for the RV dysfunction cases may contribute to the clinical observation of exercise intolerance in HFpEF patients. Stable HFpEF subjects can present as normal at rest during clinical evaluations with symptoms such as impaired LV filling only becoming apparent during exertion (Borlaug et al., 2010; Dunlay, Roger, and Redfield, 2017). Knight et al. reported a link between RV dilation and LV DD in patients with RV dysfunction due to PH (Knight et al., 2015); however it was unclear whether this linkage was due to abnormal septal dynamics, pericardial constraints, or intrinsic myocardial stiffening of the LV. As discussed in Section 4.3, our simulations suggest that LV DD in RV dysfunction results from VVI mediated by the septum. Along with reports of substantial numbers of HFpEF patients exhibiting RV SD or DD, our *in silico* findings suggest that a subset of HFpEF patients may not have primary LV failure, but in fact primary RV failure (Rommel et al., 2018). Moreover, Supplementary Figure S1 shows no substantial change in CPO as HR increases in these dysfunction cases. This interpretation offers an explanation for the heterogeneity of the HFpEF diagnosis well known clinically (Dunlay, Roger, and Redfield, 2017) and recently investigated using model-based analysis and physiology-informed machine learning (Jones et al., 2021).

### 4.5 Subject-specific parameterization

In this study, we developed a systematic method to calculate parameters for the TriSeg model given input data (Table 1). Subject-specific modeling has gained momentum in recent years because of its potential to aid in the diagnosis and management of disease that is tailored to the patient, creating a “digital twin” (Corral-Acero et al., 2020) or a virtual representation of an individual’s overall health. Thus, various modeling frameworks have employed subject-specific methods (Krishnamurthy et al., 2013; Miller et al., 2021). Here, cardiac and vascular nominal parameter sets ensure subject-specific predictions given available routinely collected clinical data, such as blood pressure, HR, LV volumes from echocardiograms, and RV and pulmonary pressures from right heart catheterization. In the future, additional measurements, such as ventricular wall thickness determined from imaging data in subjects with HR, can be used to inform model parameters *a priori*. In this study, we have parameterized the model for a general subject with the data from Table 1. However, through our methodology, virtually any subject-specific CVS measurements can be substituted for the representative data presented and used here. Moreover, we have demonstrated the ability of the model to adapt to different heart sizes by scaling EDV to directly achieve different EDPVRs (Figure 2), computationally demonstrating the phenomenon described by Klotz. Thus, we believe this model with the parameterization developed here has many potential applications in the analysis of various CVS disease states. Though other studies have proposed frameworks for parameterizing systemic and pulmonary circulations (Marquis et al., 2018; Jones et al., 2021), to our knowledge this is the first comprehensive subject-specific parameterization of the TriSeg model. Future work includes using this model in conjunction with subject data, such as exercise data in subjects with HF, to create patient-specific models and investigate these mechanisms in different disease states.

### 4.6 Limitations and future work

We propagated several assumptions from the TriSeg model including spherical ventricular geometries and sarcomere length-tension relationships from isolated rat cardiac muscles. In this study, we used a simplified circulatory system that lacks compensatory and regulatory mechanisms, such as the baroreflex (Jezek et al., 2022). However, to overcome this, we employed compensatory volume overload as a surrogate, showing similar regulatory effects. For simplicity, we have not explicitly modeled the atria but instead incorporated the volumes occupied by the left and right atria into the venous and systemic venous compartments, respectively. Thus, the atrial volume does not contribute to the overall pericardial volume in this model which leads to a slightly different behavior *in vivo*. However, the pericardium still comes into effect at higher volumes. Future work can include the incorporation of atria as well as further investigation into modeling pericardial dynamics. By modeling only forward blood flow through the heart valves, we have ignored phenomena, such as valve regurgitation (Havlenova et al., 2021), to maintain focus on the interaction between the ventricles as high pressure develops during HF. The impact of valve regurgitation in disease is an active area of research (Korakianitis and Shi, 2006; Blanco and Feijóo, 2010; Pant et al., 2016; Chabiniok et al., 2017; Sacks et al., 2019). Future work includes modeling this phenomenon in this system and its impact on VVI. More biophysically based models may be able to explain the observed phenomena in a more detailed fashion. Here we present a simple, low-order model to conduct our initial investigations, but our modeling framework allows for the flexibility to integrate with more detailed models as in (Hunter et al., 1998; Lumens et al., 2009; Niederer, Campbell, and Campbell, 2019; Beard et al., 2022). Lastly while there are limitations to this idealized subject forward-modeling approach, we reference published data and results to corroborate the model outputs and results, and the updated parameterization of the model facilitates the use of clinical data and subject-specific modeling in the future.

Further model validation routes include identification of the model data from clinical and preclinical studies. Clinical data may include right heart catheterization and echocardiograms from HFpEF patients to which the model could be fit using the approach described in Jones et al. (2021). Additionally, future experiments in which simultaneous biventricular pressure-volume loops and septal curvature data are obtained under baseline conditions, with volume loading, and during various perturbations to LV and RV free wall mechanics would provide key evidence to test the hypotheses inherent in the model.

## 5 Conclusion

In this study, we developed and parameterized a subject-specific mathematical model of cardiovascular function and biventricular mechanics to investigate several modes of heart failure, including systolic and diastolic dysfunction of the left and right ventricles. In acute LV systolic and diastolic dysfunction, the simulations captured RV compensation with systemic volume loading to maintain stroke volume and mean arterial blood pressure. In RV systolic and diastolic dysfunction, RV dilation and volume overload impaired LV filling, resulting in an LV diastolic dysfunction caused by mechanical interventricular interactions rather than LV myocardial stiffening. We also observed that three of the dysfunction simulations (LV diastolic dysfunction, RV systolic and diastolic dysfunction) exhibited phenotypes seen in HFpEF, suggesting multiple modes of heart failure in HFpEF, which is consistent with and may help to explain the clinical heterogeneity of HFpEF. More defined diagnostic sub-categories of HFpEF will allow for the development of targeted treatments and improved outcomes of this disease.

## Data Availability

The original contributions presented in the study are included in the article/Supplementary Materials, further inquiries can be directed to the corresponding authors.

## Supplementary material

The run-time environment of the model is MATLAB 2021b. A release of the model codes can be found at DOI: 10.5281/zenodo.8248116 and updates can be found at https://github.com/sallakim/ventricular-interdependence-simulations.

## Abbreviations

CPO, Cardiac power output; CVS, Cardiovascular system; DBP, Arterial diastolic blood pressure; DD, Diastolic dysfunction; EDP End-diastolic pressure; EDV, End-diastolic volume; EDPVR, End-diastolic pressure-volume relationship; ESP, End-systolic pressure; ESV, End-systolic volume; ESPVR, end-systolic pressure-volume relationship; HF, Heart failure; HFrEF, Heart failure with reduced ejection fraction; HFpEF, Heart failure with preserved ejection fraction; HFmrEF, Heart failure with mid-range ejection fraction; HR, Heart rate; LW, Left ventricular free wall; LV, Left ventricle; M, Moderate; RW, Right ventricular free wall; RV, Right ventricle; S, Severe; SBP, Arterial systolic blood pressure; SD, Systolic dysfunction; SW, Septal wall; TriSeg Three-segment biventricular model; VVI, Ventricular-ventricular interactions.
