# Supplementary material for: Computational modeling of ventricular-ventricular interactions suggest a role in clinical conditions involving heart failure
Source: Front Physiol. 2023 Sep 6;14:1231688. doi: 10.3389/fphys.2023.1231688 (PMC10512181; doi:10.3389/fphys.2023.1231688)
Supplement: Supplementary file 1 [file Presentation1.pdf]

Supplemental Appendix to Computational Modeling of Ventricular-Ventricular Interactions Suggest a Role in Clinical Conditions Involving Heart Failure

This appendix details the heart and cardiovascular model equations and parameters. The run-time environment of the model is MATLAB 2021b. A release of the model codes can be found at DOI: 10.5281/zenodo.8248116 and all updates can be found at https://github.com/sallakim/ventricular-interdependence-simulations.

Cardiac Model: Geometry

The geometry of the wall segments is represented in terms of the axial, $x_{m,i}$, and radial, y_m, midwall displacements such that

$$V_{m,i} = \frac{\pi}{6} x_{m,i}\left(x_{m,i}^2 + 3y_m^2\right), \tag{A1}$$

$$C_{m,i} = \frac{2x_{m,i}}{x_{m,i}^2 + y_m^2}, \quad \text{and} \tag{A2}$$

$$A_{m,i} = \pi\left(x_{m,i}^2 + y_m^2\right), \tag{A3}$$

where $V_{m,i}$, $C_{m,i}$, and $A_{m,i}$ are the midwall volumes, curvatures, and areas. Note that for the LW the radius of curvature has a negative value. For simplicity, the dimensionless ratio

$$z_i = \frac{3C_{m,i}V_{w,i}}{2A_{m,i}} \tag{A4}$$

is established, which is related to the ratio of wall thickness to the radius of curvature.

Cardiac Model: Sarcomere mechanics

The time-dependent sarcomere length, L_i, is modeled as a passive element in parallel with a series combination of a contractile element and series elastic element. L_i (μm) is given by

$$L_i = L_{ref}e^{\varepsilon_i}, \tag{A5}$$

where L_{ref} (μm) is the reference sarcomere length at zero strain, and ε_i is the myofiber strain formulated as

$$\varepsilon_i = 0.5 \ln\frac{A_{m,i}}{A_{m,ref,i}} - \frac{1}{12}z_i^2 - 0.019z_i^4. \tag{A6}$$

Note that L_i is L_{ref} for isometric contractions. The length of the contractile element of the sarcomere, $L_{c,i}$, is the solution to the differential equation

$$\frac{dL_{c,i}}{dt} = \left(\frac{L_i - L_{c,i}}{L_{se,iso}} - 1\right)v_{max}, \tag{A7}$$

where $L_{se,iso}$ (μm) is the isometrically stressed series elastic element length and v_{max} (μm s^{-1}) is the sarcomere shortening velocity at zero load, and both are constants (**Table 2**).

Cardiac Model: Ventricular chamber pressure

The tension ($T_{m,i}$, kPa m) developed at the midwall junction is derived from myofiber stress and wall segment geometry according to Lumens et al. given as

$$T_{m,i} = \left(\frac{V_{w_i}\sigma_i}{2A_{m_i}}\right)\left(1 + \frac{z_i^2}{3} + \frac{z_i^4}{5}\right), \tag{A8}$$

where σ_i (kPa) is the total stress (**Equation 1**). The axial ($T_{x,i}$, kPa m) and radial ($T_{y,i}$, kPa m) midwall tension components are

$$T_{x,i} = \left(\frac{2x_{m,i}y_m}{x_{m,i}^2 + y_m^2}\right)T_{m,i} \quad \text{and} \tag{A9}$$

$$T_{y,i} = \left(\frac{-x_{m,i}^2 + y_m^2}{x_{m,i}^2 + y_m^2}\right)T_{m,i}. \tag{A10}$$

By the Law of Laplace, ventricular chamber pressures are then

$$P_{LV} = -\frac{2T_{x,LW}}{y_m} \quad \text{and} \quad P_{RV} = \frac{2T_{x,RW}}{y_m}. \tag{A11}$$

Cardiac Model: Force and volume balance

The tensions in the axial and radial directions are balanced such that

$$0 = T_{k,LW} + T_{k,SW} + T_{k,RW}, \tag{A12}$$

where $k = x$ or y. The ventricular volumes are balanced such that

$$0 = -EDV_{LV} - 0.5V_{w,LW} - 0.5V_{w,SW} + V_{m,SW} - V_{m,LW} \quad \text{and} \tag{A13}$$

$$0 = EDV_{RV} - 0.5V_{w,RW} - 0.5V_{w,SW} + V_{m,SW} - V_{m,RW}. \tag{A14}$$

Circulation Model: Volume Distribution

Circulation model compartmental volumes are scaled by the TBV using blood volume fractions, d_j, based on (Jones et al. 2021; Marquis et al. 2018) and adapted from (Beneken 1979) as $V_{0,j} = d_j\text{TBV}$ where $j = LV, RV, SA, SV, PA,$ and PV. Blood volume fractions are listed in **Table 3**. The volume in the four vascular compartments ($SA, SV, PA,$ and PV) is the sum of the unstressed ($V_{u,j}$, mL) and stressed ($V_{s,j}$, mL) volumes as $V_{0,j} = V_{u,j} + V_{s,j}$, where the unstressed volume is the maximal volume of blood for which the compartment experiences zero pressure on its wall and the stressed volume is the volume beyond the unstressed volume to raise compartment pressure above zero. The stressed volume is a percentage of the unstressed volume, that is, $V_{s,j} = b_j V_{u,j}$. Unstressed volume ratios are listed in **Table 3**.

Circulation Model: Chamber Pressure Scaling

We use nominal healthy pressures (denoted by a hat) from (Boron 2016) for each model compartment (listed in **Table 3**) which are used in the calculation of resistances and compartmental compliances and chamber elastances. To ensure the model predictions are subject-specific, first we use the chamber data from **Table 1** and let $\hat{P}_{m,LV} = \text{EDP}_{LV}$, $\hat{P}_{M,LV} = \text{ESP}_{LV}$, $\hat{P}_{m,RV} = \text{EDP}_{RV}$, and $\hat{P}_{M,RV} = \text{ESP}_{RV}$. Then we scale the circulation model compartments by the nominal healthy systemic systolic ($\hat{P}_{M,SA} = 120$ mmHg) diastolic ($\hat{P}_{m,SA} = 80$ mmHg) blood pressures, that is, minimum ($n_{m,j}$) and maximum ($n_{M,j}$) blood pressure scalars are

$$n_{M,j} = \frac{\hat{P}_{M,j}}{\hat{P}_{M,SA}} \text{ and } n_{m,j} = \frac{\hat{P}_{m,j}}{\hat{P}_{m,SA}}. \tag{A15}$$

Compartmental nominal pressures are recapitulated given systemic arterial pressure data ($\overline{\text{SBP}}$ and $\overline{\text{DBP}}$) as in **Table 1**, so

$$\hat{P}_{M,j} = n_{M,j}\,\overline{\text{SBP}} \quad \text{and} \quad \hat{P}_{m,j} = n_{m,j}\,\overline{\text{DBP}}. \tag{A16}$$

Circulation Model: Compliance and Resistance

We approximate compartmental compliances (C_j, mL mmHg^{-1}) and intercompartmental resistances (R_j, mmHg s mL^{-1}) based on stressed volumes and maximum pressures. That is, C_j is the ratio of the maximal compartmental stressed volume ($V_{i,s}$) to the estimated systolic compartmental pressure ($P_{M,j}$, mmHg)

$$C_j = \frac{V_{s,j}}{P_{M,j}}. \tag{A17}$$

R_j is the ratio of the pressure drop across the resistance to the CO. Here, the pressure drop is defined as the difference between the max systolic pressure in the arterial compartment preceding the resistance ($P_{M,j}$, mmHg) and the mean pressure (denoted by a bar) from the venous compartment following the resistance ($P_{m,j+1}$, mmHg), i.e.,

$$R_j = \frac{P_{M,j} - P_{bar,j+1}}{\text{CO}}. \tag{A18}$$

The mitral and tricuspid valve resistances are similarly computed as a pressure difference but is instead the pressure difference is between the mean pressure of the preceding venous compartment and the minimum resistance of the following ventricular compartment.

Circulation Model: Model Equations

The circulation model is formulated such that the circulating volume is the sum of the stressed volume for all compartments. Volume is conserved by formulating differential equations using Kirchhoff's law as

$$\frac{\mathrm{d}V_{s,j}}{\mathrm{d}t} = Q_{in} - Q_{out}, \tag{A19}$$

where Q_{in} and Q_{out} are the time-dependent flows in and out of each compartment. For the arterial compartments, the chamber pressure has a linear relationship with its chamber volume with the compliance (C_j, mL mmHg^{-1}) and transmural resistance ($R_{t,j}$, mmHg s mL^{-1}), defined above, as

$$P_{SA} = \frac{V_{s,SA}}{C_{SA}} + (Q_a - Q_{SA})R_{t,SA} \tag{A20}$$

where Q_a and Q_{SA} are the blood flows across the aortic valve and systemic resistance, respectively. The remaining vascular compartments have linear pressure-volume relationships given by

$$P_{PA} = \frac{V_{s,PA}}{C_{PA}},\ P_{SV} = \frac{V_{s,SV}}{C_{SV}}, \text{ and } P_{PV} = \frac{V_{s,PV}}{C_{PV}}. \tag{A21}$$

Flows across the systemic and pulmonary resistances are formulated using Ohm's Law as

$$Q_{SA} = \frac{P_{SA} - P_{SV}}{R_{SA}} \quad \text{and} \quad Q_{PA} = \frac{P_{PA} - P_{PV}}{R_{PA}}, \tag{A22}$$

where R_{SA} (mmHg s mL^{-1}) and R_{PA} (mmHg s mL^{-1}) are the systemic and pulmonary resistances, respectively, and Q_{PA} (mL s^{-1}) is the pulmonary arterial flow. Flows through the aortic (Q_a, mL s^{-1}), mitral (Q_m, mL s^{-1}), pulmonary (Q_p, mL s^{-1}), and tricuspid (Q_t, mL s^{-1}) valves are modeled as diodes such that only forward flow through the valves is permitted, and the valves open and close instantaneously given a threshold pressure as

$$Q_l = \max\left(\frac{P_{in} - P_{out}}{R_l}, 0\right), \tag{A23}$$

for $l = m, a, t,$ or p and R_l (mmHg s mL^{-1}) is the resistance of the valve. Transmural and valve resistance parameters are listed in **Table 2**.

Derivation of Γ

In **Equations 11** and **12** we calculate the chamber pressure, P_i, in terms of the wall stress, σ_i, and Γ, which is a term that encompasses the geometric relationship, z_i, and summarizes the relationship of the chamber

pressure to the wall stress. To derive this, we use the Laplace's Law, which states that at a given ventricular chamber pressure (P_i), we have

$$\frac{P_i\, y_m}{2} = T_{x,i}. \tag{A24}$$

Substituting **Equations A8 and A9**,

$$\frac{P_i\, y_m}{2} = \frac{2\, x_{m,i}\, y_m}{x_{m,i}^2 + y_m^2} \left(\frac{V_{w,i}}{2\, A_{m,i}} \left(1 + \frac{1}{3} z_i^2 + \frac{1}{5} z_i^4\right) \sigma_i \right).$$

Substituting **Equation A2**,

$$\frac{P_i\, y_m}{2} = \; C_m\, y_m \left(\frac{V_{w,i}}{A_{m,i}} \left(1 + \frac{1}{3} z_i^2 + \frac{1}{5} z_i^4\right) \sigma_i \right).$$

Combining terms and substituting **Equation A4**, we have

$$\frac{P_i\, y_m}{2} = \; -\frac{1}{3}\, z_i\, y_m \left(1 + \frac{1}{3} z_i^2 + \frac{1}{5} z_i^4\right) \sigma_i.$$

Finally, eliminating y_m and solving for pressure yields

$$P_i = \; -\frac{2}{3}\, z_i \left(1 + \frac{1}{3} z_i^2 + \frac{1}{5} z_i^4\right) \sigma_i. \tag{A25}$$

Then,

$$\Gamma = \; -\frac{2}{3}\, z_i \left(1 + \frac{1}{3} z_i^2 + \frac{1}{5} z_i^4\right). \tag{A26}$$

Klotz et al. Single-Beat Estimation of the EDPVR

From (Klotz et al. 2006), given a single-beat measurement of the EDP and EDV, the EDPVR is approximated as

$$\text{EDP} = \; \alpha\, \text{EDV}^{\beta}, \tag{A27}$$

where

$$\alpha = \frac{30}{{V_{30}}^{\beta}} \quad \text{and} \quad \beta = \frac{\ln\left(\frac{\text{EDP}}{30}\right)}{\ln\left(\frac{\text{EDV}}{V_{30}}\right)}. \tag{A28}$$

for V_0 (mL) and V_{30} (mL) the volumes at which the EDP is ~0 and 30 mmHg, respectively. To approximate V_0 and V_{30}, we have

$$V_0 = \text{EDV}\,(0.6 - 0.006\,\text{EDP}) \tag{A29}$$

and

$$V_{30} = V_0 + \frac{\text{EDV} - V_0}{\left(\frac{\text{EDP}}{A_n}\right)^{1/B_n}}, \tag{A30}$$

where $A_n = \; 28$ (mmHg) and $B_n = 3$ (unitless).

The EDVs were normalized as follows for the normalized EDPVR

$$\text{EDV}_n = \frac{(\text{EDV} - V_0)}{(V_{30} - V_0)}. \tag{A31}$$

References

Beneken, J. E. W. 1979. *A physical approach to hemodynamic aspects of the human cardiovascular system* (W. B. Saunders: PA, USA).

Boron, Walter; Boulpaep, Emile. 2016. *Medical Physiology 3rd Edition* (ELSEVIER: Philadelphia, PA).

Jones, E., E. B. Randall, S. L. Hummel, D. M. Cameron, D. A. Beard, and B. E. Carlson. 2021. 'Phenotyping heart failure using model-based analysis and physiology-informed machine learning', *J Physiol*, 599: 4991-5013.

Klotz, S., I. Hay, M. L. Dickstein, G. H. Yi, J. Wang, M. S. Maurer, D. A. Kass, and D. Burkhoff. 2006. 'Single-beat estimation of end-diastolic pressure-volume relationship: a novel method with potential for noninvasive application', *Am J Physiol Heart Circ Physiol*, 291: H403-12.

Marquis, Andrew D., Andrea Arnold, Caron Dean-Bernhoft, Brian E. Carlson, and Mette S. Olufsen. 2018. 'Practical identifiability and uncertainty quantification of a pulsatile cardiovascular model', *Mathematical Biosciences*, 304: 9-24.
