# Supplementary material for: Computational modeling of ventricular-ventricular interactions suggest a role in clinical conditions involving heart failure
Source: Front Physiol. 2023 Sep 6;14:1231688. doi: 10.3389/fphys.2023.1231688 (PMC10512181; doi:10.3389/fphys.2023.1231688)
Supplement: Supplementary file 2 [file DataSheet1.pdf]

Supplemental Material to Computational Modeling of Ventricular-Ventricular Interactions Suggest a Role in Clinical Conditions Involving Heart Failure

Healthy and dysfunction cases were simulated for different heart rates: 60, 80, and 100 bpm. The cardiac power output at full compensation (i.e., performed at a mean systemic arterial pressure of ~95 mmHg) is shown in **Figure S1**. If full compensation was not achieved, it was excluded from the plots. Values are shown in **Table S1**.

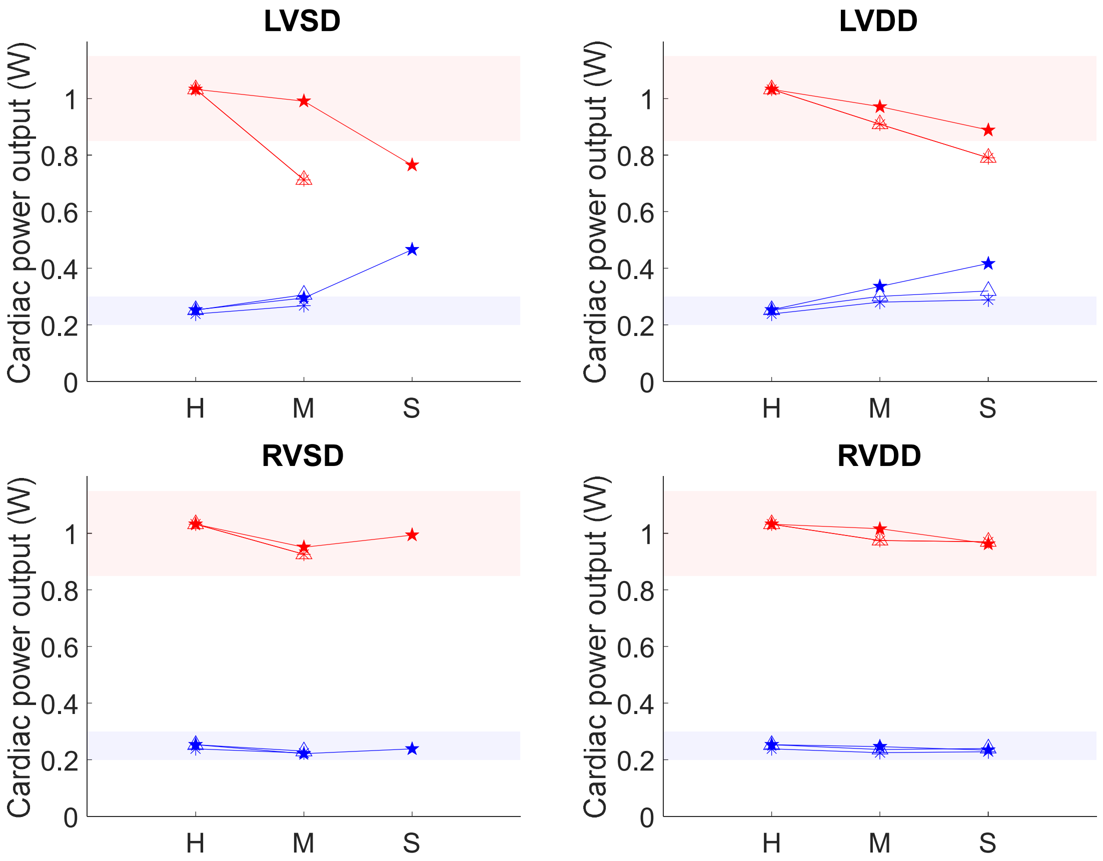

Figure S1: Cardiac power output for the left (LV, red) and right (RV, blue) ventricles for heart rates at 60 (star), 80 (triangle), and 100 (asterisk) bpm. SD – systolic dysfunction. DD – diastolic dysfunction.

Table S1: Cardiac power output (W) at several heart rates.

	**HR = 60**		**HR = 80**		**HR = 100**	
	**LV**	**RV**	**LV**	**RV**	**LV**	**RV**
Healthy	1.04	0.25	1.03	0.25	0.98	0.24
LVSD, M	0.99	0.30	0.71	0.31	0.61	0.27
LVSD, S	0.76	0.47	-	-	-	-
LVDD, M	0.97	0.34	0.91	0.30	0.87	0.28
LVDD, S	0.89	0.42	0.79	0.32	0.75	0.29
RVSD, M	0.95	0.22	0.93	0.23	0.89	0.22
RVSD, S	0.99	0.24	-	-	-	-
RVDD, M	1.02	0.25	0.97	0.24	0.93	0.22
RVDD, S	0.96	0.23	0.97	0.24	0.93	0.23

LV – Left ventricle. RV – Right ventricle. SD – Systolic dysfunction. DD – Diastolic Dysfunction.
